# Hepatocellular Carcinoma and Health-Related Quality of Life: A Systematic Review of Outcomes From Systemic Therapies

**DOI:** 10.1155/ijh/1083642

**Published:** 2025-04-07

**Authors:** Dujinthan Jayabalan, Sugam Dhakal, Aarohanan Raguragavan, Akshat Saxena, Gary P. Jeffrey, Luis Calzadilla-Bertot, Leon A. Adams, Michael C. Wallace

**Affiliations:** ^1^Department of Hepatology, Sir Charles Gairdner Hospital, Nedlands, Western Australia, Australia; ^2^Medical School, The University of Western Australia, Nedlands, Western Australia, Australia; ^3^Department of Cardiothoracic Surgery, Fiona Stanley Hospital, Murdoch, Western Australia, Australia

**Keywords:** atezolizumab/bevacizumab, health-related quality of life, hepatocellular carcinoma, lenvatinib, sorafenib, systematic review, transarterial radioembolisation

## Abstract

**Aim:** Poor outcomes in advanced hepatocellular carcinoma (HCC) coupled with potential significant treatment side effects underpin a strong rationale to assess health-related quality of life (HRQOL) in those treated with systemic therapies. This study is aimed at quantifying the effect of systemic therapies on HRQOL outcomes in HCC patients when compared to baseline or placebo, other systemic therapies, and transarterial radioembolisation (TARE).

**Methods:** In May 2024, two independent reviewers searched PubMed, EMBASE, and Google Scholar for studies comparing postsystemic therapy HRQOL scores in adult patients with HCC to baseline or placebo, other systemic therapies, or to TARE. Narrative synthesis was used to synthesise results. Risk of bias was assessed using RoB 2 and ROBINS-I. This review was structured according to PRISMA guidelines and was prospectively registered in the PROSPERO register (CRD42024521699).

**Results:** Twenty-nine studies with 10,472 patients using eight HRQOL instruments were included. Compared to baseline, patients on atezolizumab/bevacizumab and sorafenib both experienced significant declines in HRQOL, and lenvatinib nonsignificantly decreased HRQOL. HRQOL remained unchanged in patients on pembrolizumab or nivolumab. Atezolizumab/bevacizumab and lenvatinib both significantly delayed HRQOL deterioration compared to sorafenib. Compared to TARE, atezolizumab/bevacizumab delayed time-to-deterioration in HRQOL, whereas sorafenib had significantly worse HRQOL.

**Conclusion:** Despite worsening HRQOL outcomes compared to baseline, the first-line agents atezolizumab/bevacizumab and lenvatinib had superior HRQOL outcomes in comparison to sorafenib. Sorafenib significantly worsened HRQOL compared to TARE. As the majority of included studies included sorafenib, which has been largely superseded by newer therapies, further trials evaluating HRQOL with these newer therapies are required.

## 1. Introduction

Hepatocellular carcinoma (HCC) is the most common primary liver cancer and the fourth most common cause of cancer-related mortality worldwide [1]. The prognosis of HCC is poor, and mortality remains high due to limited systemic treatment options and delayed diagnosis resulting from failed early detection [2]. Surgical resection is commonly not possible when HCC is diagnosed due to advanced tumour stage or severe underlying liver disease [3].

Systemic therapies (immune checkpoint inhibitors, tyrosine kinase inhibitors, and monoclonal antibodies) are considered first-line therapy for advanced HCC [4]. The tyrosine kinase inhibitor sorafenib was the first systemic therapy for HCC to demonstrate a survival benefit [5]. With recent advancements, systemic options have since expanded to include a myriad of agents such as atezolizumab/bevacizumab (atezo/bev), lenvatinib, pembrolizumab, nivolumab, ramucirumab, cabozantinib, dovitinib, brivanib, regorafenib, and durvalumab-tremelimumab, with atezo/bev and lenvatinib representing the most commonly used first-line options [4, 6]. Transarterial radioembolisation (TARE) is an effective locoregional treatment option considered in those with advanced HCC [7, 8]. Despite a vast array of treatment alternatives, patients with advanced HCC continue to experience poor clinical outcomes and limited overall survival [9, 10].

Poor outcomes experienced by patients with advanced HCC underpin the importance of measuring the impact of treatment on health-related quality of life (HRQOL). HRQOL is defined by the US Food and Drug Administration as “a multidomain concept that represents the patient's overall perception of the impact of an illness and its treatment” [11]. Despite increasing overall survival in patients with advanced HCC, systemic therapies have a wide adverse reaction profile, including nausea and vomiting, diarrhoea, fatigue, hypertension, proteinuria, haematological toxicities, and palmar-plantar erythrodysesthesia, posing a significant detriment to patients' HRQOL [12–14].

Given the recent advances in the management of advanced HCC, it is important that HRQOL is captured from clinical trials and postmarketing studies and reported to ensure optimal guidance of clinician judgement in the management of patients with HCC on systemic therapy. This systematic review aimed to (i) summarise the literature on and to demonstrate and quantify the effect of systemic therapies on HRQOL outcomes in HCC patients when compared to baseline or placebo, other systemic therapies, and to TARE and (ii) provide a foundation for future research into HRQOL in HCC patients on systemic therapies.

## 2. Materials and Methods

This systematic review was structured according to the Preferred Reporting Items for Systematic Reviews and Meta-Analyses (PRISMA) guidelines (Tables S1 and S2) [15, 16] and aligns with the methodology outlined by Grant and Booth [17]. This systematic review was prospectively registered in the international prospective register of systematic reviews (PROSPERO) register (CRD42024521699) and followed the PRISMA and PROSPERO guidelines.

### 2.1. Measurement of HRQOL

Validated generic HRQOL instruments in patients with liver disease incorporated in this review include the Medical Outcomes Survey Short-Form 36 (SF-36) [18], EuroQol 5D (EQ-5D) [19], and the Quality of Life Questionnaire Core 30 (QLQ-C30) [20, 21]. The cancer-specific instrument, Functional Assessment of Cancer Therapy–General Questionnaire (FACT-G) was also included [22]. Validated, liver disease-specific questionnaires used in HCC studies in this systematic review include the Quality of Life Questionnaire Hepatocellular Carcinoma (QLQ-HCC18) [23], the Functional Assessment of Cancer Therapy–Hepatobiliary Questionnaire (FACT-Hep) [21, 24], the Functional Assessment of Cancer Therapy–Hepatobiliary Subscale (FACT-HS) [25], and the Functional Assessment of Cancer Therapy Hepatobiliary Cancer Symptom Index (FHSI-8) [26].

### 2.2. Eligibility Criteria

All studies included in the current review were original manuscripts published in peer-reviewed journals. Study designs meeting eligibility criteria included randomised-control trials (RCTs), nonrandomised trials, cohort studies, cross-sectional studies, and case series. Systematic reviews, meta-analyses, letters to the editor, case reports, and conference abstracts were excluded. Any clinical trial published in English evaluating the effects of systemic therapies on HRQOL in adult (≥ 18 years) patients with HCC conducted after the year 2000 was included. Studies were required to meet the following characteristics for inclusion: (1) adult HCC patients on systemic therapies (immune checkpoint inhibitors, tyrosine kinase inhibitors, and monoclonal antibodies), (2) validated disease-specific or generic HRQOL instruments utilised to present HRQOL data, and (3) post-treatment scores compared to baseline or placebo, other systemic therapies, or TARE. Further inclusion criteria required (1) date of publication after January 2000, (2) English language, and (3) original articles.

### 2.3. Information Sources and Search Strategy

**Figure 1:**
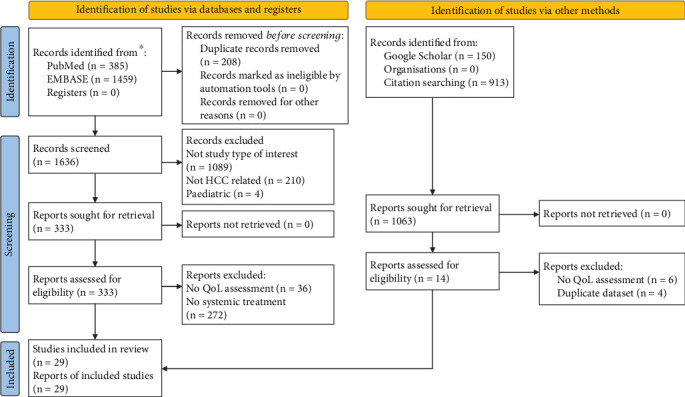
PRISMA flow diagram of included studies.

In May 2024, using the previous eligibility criteria, a preliminary search of the literature using Medical Subject Headings (MeSH) keywords was conducted on PubMed and EMBASE (Figure 1). To identify studies not identified in the preliminary MeSH keyword search, a manual search of Google Scholar, MEDLINE (Ovid), and a review of all included study bibliographies was completed. The aforementioned databases were used for the retrieval of all identified articles. The search strategy has been recorded (Table S3).

### 2.4. Study Selection

Following the search, D.J. and S.D. independently screened titles and abstracts of all studies retrieved. Duplicates were removed manually. Studies that had titles and abstracts that met inclusion criteria or had inadequate information to determine exclusion or inclusion underwent independent full-text review for eligibility by D.J. and S.D. Studies were not included if they did not meet eligibility criteria. Kappa statistics were used to verify interinvestigator reliability [27]. Based on the predetermined eligibility criteria, disagreements were resolved by consensus between D.J., S.D., A.R., and M.C.W. The study selection eligibility process is illustrated in a PRISMA flow diagram (Figure 1).

### 2.5. Data Items and Extraction

Three prepiloted, data extraction forms were developed by D.J., A.R., and A.S., one for assessment of baseline characteristics (Table 1), one for key findings (Table 2), and one for complete study results (Table S4). For each included study, Table 1 includes year of publication, author, number of patients, study design, HRQOL instrument used (generic and disease-specific), participation rate, response rate, the National Health and Medical Research Council (NHMRC) level of evidence, and the following patient demographics: age, sex, location, aetiology of HCC, and HCC staging. For each included study, Table 2 includes the method of follow-up, the follow-up time intervals, the comparison groups (baseline or placebo, other systemic therapies, or TARE), and key findings. Data extraction was performed by D.J. and S.D. independently, with disagreements resolved by consensus between D.J., S.D., and A.R. Results were grouped by comparison to baseline or placebo, to systemic therapies, and to TARE for easier comparison and analysis.

### 2.6. Synthesis of Results

In accordance with previous guidelines, qualitative analysis was performed through narrative synthesis where HRQOL outcomes were categorised into physical, emotional, social, and functional health domains, which were either disease-specific or generic [18–26, 28].

### 2.7. Risk of Bias

Risk of bias was assessed in individual study data using qualitative analysis based on study quality. Included studies were evaluated for significant selection, performance, detection, or reporting bias, according to the Cochrane guidelines on systematic reviews [29]. Assessment of bias was carried out in accordance with PRISMA guidelines [15, 16]. The NHMRC Evidence Hierarchy, used to assess the level of evidence of individual studies, participation rates (the proportion of patients answering at least one question in a HRQOL questionnaire), and response rates (the proportion of patients who completed all questions in all HRQOL questionnaires), is outlined in Table 1 [30].

For RCTs, risk of bias was evaluated using the revised Cochrane risk-of-bias tool for randomised trials (RoB 2) [31]. The risk of bias of nonrandomised, interventional trials was assessed with the ROBINS-I tool (risk of bias in nonrandomised studies of interventions, previously known as ACROBAT-NRSI) as recommended by the Cochrane collaboration [32]. Case series were assessed using the Canada Institute of Health Economics (IHE) quality appraisal checklist [33]. RCTs were judged to be at an overall high risk of bias if there was a serious risk in any of the following domains: randomisation process, deviation from intended intervention, missing outcome data, measurement of outcome, and selection of reported result. For nonrandomised trials, a high risk of bias was concluded if there was a serious risk in any of the following domains: confounding, participant selection, classification of interventions, deviation from intended intervention, missing data, measurement of outcomes, and selection of reported result. Risk of bias assessments were performed by D.J. and S.D. independently, with disagreements resolved by consensus between D.J., S.D., and A.R.

Due to the high heterogeneity between studies, meta-analysis was not viable, and as such, specific tools were not utilised to assess bias within individual studies.

## 3. Results

### 3.1. Study Selection

One thousand eight hundred and forty-four studies were identified by database search, 208 of which were duplicates (Figure 1). After full-text review, 1636 studies were excluded, with reasons for exclusion outlined in Figure 1. An additional 308 studies were excluded after full-text review, with reasons for exclusion outlined in Figure 1. The 29 remaining studies assessing 10,472 patients were included in the final qualitative synthesis [34–62]. Kappa statistics for agreement between the reviewers on inclusion of trials were 0.95 (95% confidence interval: 0.88–1.00). The included studies exhibited diversity between HRQOL instruments utilised, comparison groups, aetiologies, stage of disease, and intermittently failed to present data as mean ± standard deviation. The presence of clinical and methodological heterogeneity precluded direct comparison through meta-analysis.

### 3.2. Study Characteristics and Risk of Bias Within Studies

**Figure 2:**
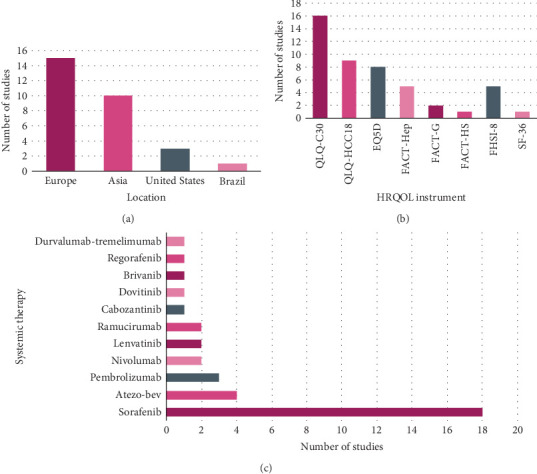
Characteristics of included studies: (a) study location, (b) HRQOL instrument, and (c) systemic therapy.

Baseline characteristics of the 29 included studies are listed in Table 1 [34–62]. Studies from 14 different countries were included, with 15 (52%) from Europe [34–36, 38, 39, 42, 43, 45–47, 56, 58–61], 10 (34%) from Asia [40, 41, 44, 48–52, 54, 62], three (10%) from the United States [53, 55, 57], and one (3%) from Brazil [37] (Figure 2a). Sixteen studies (55%) were RCTs, 10 (34%) were prospective cohort studies [36, 37, 39–41, 48, 49, 51, 54, 59], two (7%) were retrospective cohort studies [42, 43], and one (3%) study was a case series [38].

Eight different HRQOL instruments were assessed in the included studies. The generic HRQOL questionnaires SF-36 and EQ-5D were utilised in one (3%) study [40] and five (17%) studies [41, 44, 56–58, 61], respectively. The cancer-specific instruments QLQ-C30 and FACT-G were used in 16 (55%) studies [34–37, 42, 43, 45–50, 53–55, 59] and two (7%) studies [39, 61], respectively. The liver disease-specific instruments QLQ-HCC18, FACT-Hep, FACT-HS, and FHSI-8 were used in nine (31%) [34, 36, 37, 45, 47, 49, 50, 54, 59], five (17%) [39, 51, 52, 61, 62], one (3%) [60], and five (17%) [38, 39, 57, 58, 62] studies, respectively (Figure 2b).

With regard to systemic therapies, 18 (62%) studies assessed sorafenib [34–41, 43–47, 52, 53, 55, 60, 62] and four (14%) studies assessed atezo/bev [34, 42, 54, 55]. Of the remaining studies, three (10%) studies investigated pembrolizumab [48–50], two (7%) studied nivolumab [51, 52], two (7%) studied lenvatinib [45, 49], two (7%) studied ramucirumab [57, 58], and one study each evaluated cabozantinib [56], dovitinib [59], brivanib [46], regorafenib [61], and durvalumab-tremelimumab [53] (Figure 2c). Four studies compared HRQOL outcomes between sorafenib and TARE [35, 42–44].

**Figure 3:**
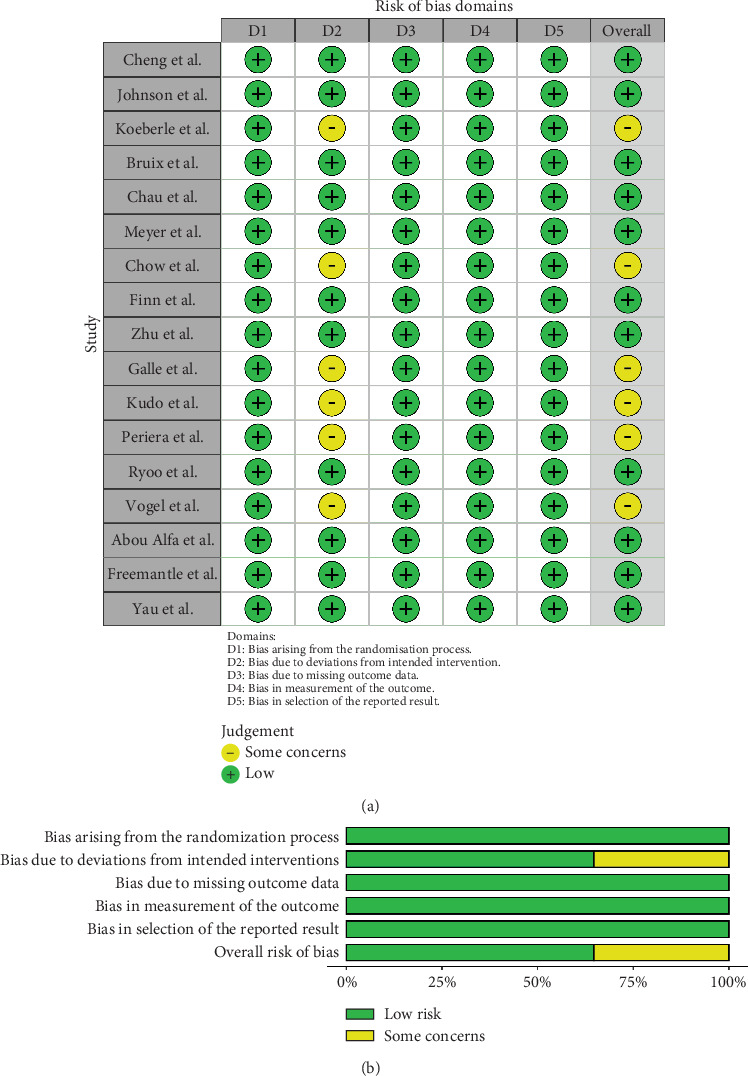
Risk of bias assessment of included randomised trials: (a) RoB 2 traffic light diagram and (b) RoB 2 summary weighted bar plot.

**Figure 4:**
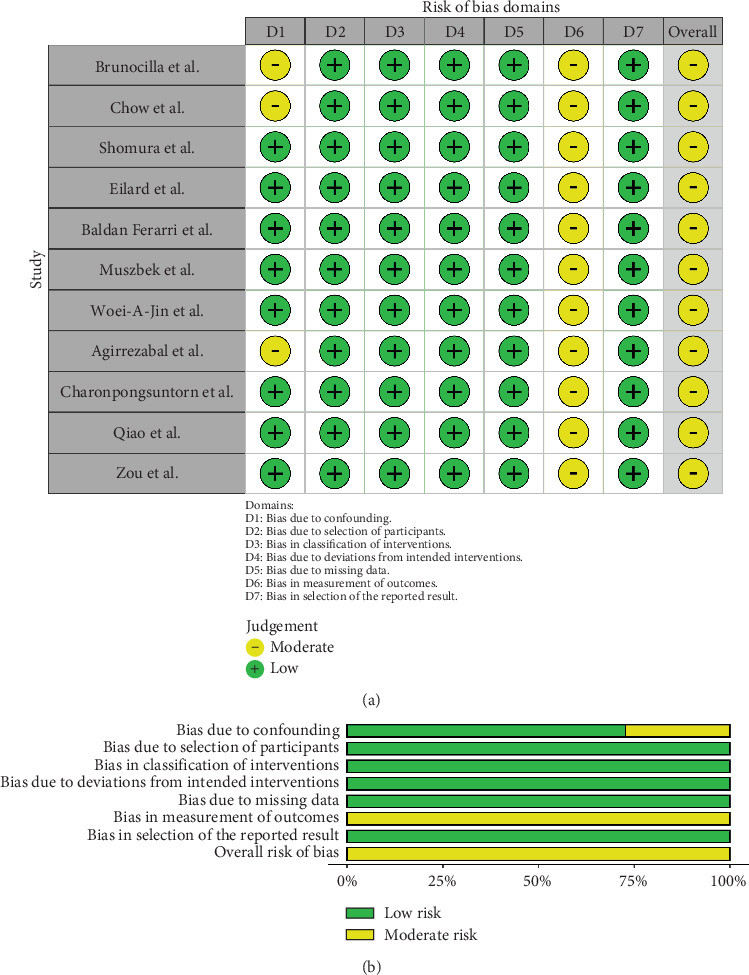
Risk of bias assessment of included nonrandomised trials: (a) ROBINS-I traffic light diagram and (b) ROBINS-I summary weighted bar plot.

Overall, no major risks were identified in the assessment of bias. Some concerns regarding bias due to deviations from the intended intervention were identified in six RCTs [34, 35, 44, 45, 51, 60] (Figure 3a,b). All 11 nonrandomised trials were judged as having a moderate risk of bias, with concerns raised in the domain measurement of outcomes in all studies [36, 37, 39–43, 48, 49, 54, 59] (Figure 4a,b). The one case series was found to have a low risk of bias [38] (Table S5).

### 3.3. Systemic Therapies Versus Baseline

Twelve studies (41%) compared HRQOL outcomes in non-sorafenib agents to baseline or placebo. Charonpongsuntorn et al. found a significant decline in EQ-5D, QLQ-C30, and in QLQ-HCC18 after 3 months of treatment with atezo/bev [54]. Kudo et al. found that nivolumab resulted in no significant decrease in FACT-Hep scores [51]. Qiao et al. and Ryoo et al. found that patients on pembrolizumab had no significant difference in QLQ-C30 scores [48, 50]. Zou et al. found significant improvements in QLQ-C30 and QLQ-HCC18 domains in patients on the programmed cell death protein 1 (PD-1) inhibitors, pembrolizumab or toripalimab, plus lenvatinib [49]. Vogel et al. reported a decrease in HRQOL scores compared to baseline in patients on lenvatinib monotherapy and sorafenib monotherapy, although significance was not reported [45].

Both Zhu et al. and Chau et al. found no significant reduction in EQ-5D and FHSI-8 scores in patients on ramucirumab [57, 58]. Additionally, Chau et al. reported that in patients with an alpha fetoprotein level of ≥ 400 ng/mL, patients on ramucirumab had superior FHSI-8 scores when compared to placebo, although both groups experienced HRQOL deterioration [58]. Freemantle et al. found that patients on cabozantinib had a significant reduction in their EQ-5D scores [56]. Johnson et al. found that brivanib led to a significant decline in the physical function and role function domains of QLQ-C30 [46]. Dovitinib significantly decreased QLQ-C30 and QLQ-HCC18 scores in Woei et al. [59]. Time-adjusted area under the curve analysis in Bruix et al. found significant reductions in FACT-Hep and EQ-5D in patients on regorafenib [61].

Seven studies primarily compared HRQOL outcomes in sorafenib to baseline or placebo. Three studies using the generic HRQOL instrument, QLQ-C30, and the HCC-specific questionnaire, QLQ-HCC18, reported significant worsening in HRQOL outcomes in patients on sorafenib compared to baseline [36, 37, 47]. The generic instruments EQ-5D and SF-36 were used in one study each to compare sorafenib to baseline or placebo [40, 41]. Shomura et al. found 24% of patients who took sorafenib for at least 1 year did not see any significant decline in their overall SF-36 scores [40]. In patients taking sorafenib 14 days postradioembolisation therapy, Chow et al. found the EQ-5D score improved over time post treatment [41]. Upon stratification according to the Barcelona Clinic Liver Cancer (BCLC) staging system, BCLC Stage B patients experienced a worsening in EQ-5D over time, while BCLC Stage C patients experienced an improvement in HRQOL [41]. Three studies used the FHSI-8 instrument to compare sorafenib to baseline or placebo, of which two studies also used FACT-Hep and one study additionally used FACT-G [38, 39, 62]. Brunocilla et al. found the FACT-G, FACT-Hep, and FHSI-8 total scores worsened at 1 week, 1 month, and 2 months post-sorafenib treatment, but the only significant worsening noted was at Week 1 [39]. Cheng et al. (using FHSI-8 and FACT-Hep questionnaires) and Montella et al. (using the FHSI-8 questionnaire) both found no significant difference in HRQOL post-sorafenib treatment [38, 62].

### 3.4. Comparison of Systemic Therapies

Two studies compared HRQOL outcomes in those treated with atezo/bev with sorafenib [34, 55]. Finn et al. found atezo/bev significantly delayed HRQOL deterioration when compared to sorafenib using the QLQ-C30 questionnaire [55]. Galle et al. also found a greater deterioration in HRQOL in sorafenib when compared to atezo/bev using both the QLQ-C30 and QLQ-HCC18, but significance was not reported [34].

Using the QLQ-C30, QLQ-HCC18, and EQ-5D questionnaires, Vogel et al. found HRQOL deterioration to be less pronounced in lenvatinib when compared to sorafenib, although there was no statistical significance [45]. Zou et al. found the addition of PD-1 inhibitors to lenvatinib significantly improved HRQOL in the QLQ-C30 domain diarrhoea and in the QLQ-HCC18 domains fatigue and pain when compared to lenvatinib monotherapy [49].

Johnson et al. found a statistically significant decline in QLQ-C30 scores of patients on brivanib and sorafenib, but no statistically significant difference between the two treatment groups was reported [46]. Yau et al. found a minimal difference in FACT-Hep and EQ-5D components to favour nivolumab over sorafenib, with nivolumab having substantially delayed HRQOL [52]. Patients on everolimus in addition to sorafenib had a nonsignificant worsening in the FACT-HS scores compared to sorafenib alone in Koeberle et al. [60]. Abou-Alfa et al. found sorafenib to have a worse time TTD in HRQOL compared to durvalumab and single tremelimumab regular interval durvalumab (STRIDE) [53].

### 3.5. Systemic Therapies Versus TARE

Three studies compared HRQOL outcomes post-sorafenib treatment with outcomes post-TARE [35, 43, 44]. Pereira et al. and Muszbek et al. used the QLQ-C30 questionnaire, while Chow et al. used the EQ-5D instrument [35, 43, 44]. Pereira et al. found that TARE treatment was associated with a significantly reduced risk in deterioration in the domains social functioning, physical functioning, role functioning, and quality of life (QOL). Despite the other domains lacking significance, the TTD for patients on sorafenib was shorter. Overall, TARE treatment displayed a reduced risk in TTD of QOL compared to sorafenib [35]. Muszbek et al. found that patients who received TARE in the SARAH trial had significantly improved global health status in the QLQ-C30 questionnaire when compared to the sorafenib group [43]. On the other hand, Chow et al. found no significant difference in EQ-5D scores between patients treated with TARE and those with sorafenib [44].

Agirrezabal et al. compared patients on atezo/bev with patients treated with TARE and patients on sorafenib as a comparison. The sorafenib group resulted in the shortest TTD in HRQOL when compared to the other two treatment groups in both case and sensitivity analyses, achieving statistical significance [42].

## 4. Discussion

### 4.1. Summary of Evidence and Interpretation

The main findings of this systematic review regarding patients with HCC undergoing systemic therapy are (i) despite worsening HRQOL outcomes compared to baseline, the first-line agents atezo/bev and lenvatinib had superior HRQOL outcomes in comparison to sorafenib; (ii) patients treated with pembrolizumab and nivolumab experienced no change in HRQOL; and (iii) sorafenib significantly worsened HRQOL compared to TARE.

Eighteen (62%) of the included studies assessed sorafenib, which despite being the first systemic therapy to demonstrate a survival benefit in HCC, is rarely used as a first-line treatment with the development of newer agents such as atezo/bev and lenvatinib [6, 55]. This shift in treatment paradigm was largely driven by improved survival outcomes, namely, the IMbrave50 and REFLECT trials [55, 63]. The current systematic review strengthens this shift away from sorafenib by summarising the superior HRQOL outcomes in patients treated with atezo/bev and lenvatinib when compared to sorafenib. Adverse events have been demonstrated to be a key theme in QOL for patients on long-term therapies [64]. Patients treated with atezo/bev have a reduced rate of adverse events when compared to sorafenib, with sorafenib having a broad adverse effect profile, which includes, but is not limited to bleeding, cardiac ischaemia, palmar-plantar erythrodysesthesia, rash, diarrhoea, nausea, vomiting, anorexia, and fatigue [65, 66]. In contrast, no difference in adverse events have been found between lenvatinib and sorafenib in a recent meta-analysis, but lenvatinib has superior overall survival, progression-free survival, objective response rate, and disease control rate when compared to sorafenib [67]. Similar to adverse events, efficacy is a key theme in maintaining patients' QOL [64]. While pembrolizumab and nivolumab did not worsen HRQOL outcomes compared to baseline in patients with advanced HCC, the addition of lenvatinib resulted in superior HRQOL outcomes when compared to lenvatinib monotherapy [49].

Pereira et al. and Eltawil et al. highlight the importance of HRQOL as a measure in patients with advanced liver disease, given the improved HRQOL in patients undergoing TARE when compared to patients on sorafenib, despite there being no significant difference between the two groups' survival [35, 68]. Studies have demonstrated that attributes other than survival, such as the adverse event profile, can lead to patients performing risk-benefit analysis between survival and the adverse event risk when deciding their treatment modality [69, 70]. TARE has a wide array of potential adverse effects, including radiation-induced liver disease, biliary complications, postradioembolisation syndrome (PRS), gastrointestinal complications, and radiation-induced pneumonia. The adverse effects of TARE, however, have a lower reported incidence than the adverse effects of sorafenib, this and is a potential explanation for this finding [65, 71].

HRQOL is an ever-increasingly important measure in oncology for optimising clinical benefits for patients, particularly for patients with advanced disease, such as advanced HCC [68]. As such, HRQOL is becoming an increasingly described outcome in the literature regarding cancer management. For example, in patients with metastatic hormone-sensitive prostate cancer, the addition of androgen receptor signalling inhibitors to androgen deprivation therapy, a therapy with proven survival benefit, has been shown to improve HRQOL outcomes when compared to other systemic therapies [72]. Neoadjuvant chemotherapy, utilised in advanced breast cancer, has been shown to result in no worsening in HRQOL [73]. Likewise, patients on cyclin-dependent kinase 4/6 inhibitor in combination with endocrine therapy, which forms the standard of care for hormone receptor–positive/human epidermal growth factor receptor 2–negative breast cancer, maintained their HRQOL throughout therapy [74].

Unfortunately, a recurrent theme is the lack of uniformity in HRQOL evaluation, inhibiting direct comparison between therapies. A recent systematic review assessing long-term outcomes in patients with advanced non–small cell lung cancer further illustrates this lack of standardisation, which included 66 studies, of which only one study reported QOL as a primary endpoint and only 38 (57.6%) studies reported HRQOL, of which heterogeneity precluded direct comparison [75]. Most pivotal Phase 3 trials in advanced HCC management reported HRQOL outcomes and were included in the current review, but the lack of uniformity in questionnaire choice and timing precluded direct comparison [45, 48, 55, 56]. Trade-offs between survival and QOL are commonly made by patients with cancer, who inevitably may not understand differences between the myriad of treatment options [76]. This necessitates comparison between HRQOL outcomes for different systemic therapies to ensure optimal shared decision-making [76]. To enable further direct comparisons of HRQOL outcomes in oncology, which would facilitate patient-centred therapeutic decision-making, advocacy for standardisation of HRQOL measurements, instruments, and time intervals is essential [69, 70].

### 4.2. Review Limitations

A limitation of this systematic review was the clinical heterogeneity of the included studies, such as variation in HRQOL instrument and time intervals of measurement, which precluded direct comparison. Strict eligibility criteria were employed to minimise reporting bias (Figure 1). Additional limitations include heterogeneity between patients undergoing systemic therapies with regard to aetiology, age, disease staging, and prior comorbidities, further limiting direct comparison of patient cohorts.

## 5. Conclusion

In conclusion, the first-line agents atezo/bev and lenvatinib had superior HRQOL outcomes in comparison to sorafenib, despite worsening HRQOL when compared to baseline, whereas pembrolizumab and nivolumab both resulted in no change in HRQOL, and sorafenib significantly worsened HRQOL when compared to TARE. As majority of the included studies assessed sorafenib, which has been superseded by newer systemic therapies, further trials evaluating HRQOL in newer agents is required. The findings of this systematic review enable greater clinical judgement when selecting management options for patients with advanced HCC and should be considered in conjunction with clinical decision-making tailored to each patient. The current review reinforces that a concerted effort should be made to standardise HRQOL evaluation in clinical trials assessing systemic therapies in advanced HCC to enable further research to better improve these patients' QOL during their therapy.

## Figures and Tables

**Table 1:** Baseline characteristics of included studies.

**Year**	**Author**	**No. of patients**	**Study design**	**Generic HRQOL instrument**	**Specific measures**	**Participation rate** **Response rate**	**Level of evidence**	**Patient demographics**				
								**Age (** **m** **e** **a** **n** ± **SD or range)**	**Sex**	**Location**	**Aetiology (%)**	**Staging systems (%)**
2009	Cheng et al.	226	RCT	—	FHSI-8 FACT-Hep	PR: 83.4% RR: 59.4%	II	Median (range) Sorafenib: 51 (23–86) Placebo: 52 (25–79)	84.7%M	Taiwan	Sorafenib: HBV 70.7, HCV 10.7 Placebo: HBV 77.6, HCV 3.9	Sorafenib: CPC: A 97.3, B 2.7 BCLC: C 95.3 Placebo CPC: A 97.4, B 2.6 BCLC: C 96.1

2012	Brunocilla et al.	36	P	FACT-G	FHSI-8 FACT-Hep	PR: NR RR: 55.6%	III	Median (range) 67.4 (35.7–82.4)	83.3%M	Italy	Cirrhosis 91.7, HBV 12.1, HCV 51.1, EtOH 15.2, NASH 6.1, HBV+HCV 6.1, HCV+EtOH 6.1, CRYP 6.1	BCLC: B 8.3, C 91.7

2013	Johnson et al.	1155	RCT	QLQ-C30	—	PR: 69.4% RR: 69.1%	II	Median (range) Sorafenib: 60 (25–89) Brivanib: 61 (19–87)	Sorafenib: 84%M Brivanib: 84%M	United Kingdom	Sorafenib: HBV 45, HCV 21, EtOH 14 Brivanib: HBV 44, HCV 20, EtOH 18	Sorafenib: CPC: A 92, B 8 BCLC: A 5, B 17, C 78 Brivanib: CPC: A 92, B 8 BCLC: A 6, B 17, C 77

2013	Montella et al.	60	Case series	—	FHSI-8	PR: NR RR: 3.3%	IV	Median (range) 76 (70–90)	78.3%M	Italy	HBV 3, HCV 68, EtOH 2, HBV+HCV 2, HCV+EtOH 2	CPC: A 73, B 22, C 3 BCLC: B 78, C 22

2014	Chow et al.	35	P	EQ-5D	—	PR: 71.4% RR: 59.2%	III	Mean ± SD 64.6 ± 10.6	72%M	Singapore	NR	CPC: A 69, B 31 BCLC: A 38, B 62

2016	Koeberle et al.	106	RCT	—	FACT-HS	PR: NR RR: NR	II	Median (range) Sorafenib: 65 (39–82) Sorafenib+everolimus: 66 (32–83)	Sorafenib: 87%M Sorafenib+everolimus: 81%M	Switzerland	Sorafenib: HBV 17, HCV 28, EtOH 59 Sorafenib+everolimus: HBV 17, HCV 29, EtOH 42	Sorafenib: CPC: 5/6 80, 7 20 BCLC: B 30, C 70 Sorafenib+everolimus: CPC: 5/6 85, 7 15 BCLC: B 25, C 75

2016	Shomura et al.	54	P	SF-36	—	PR: NR RR: 24.1%	III	< 70: 24 ≥ 70: 30	78%M	Japan	HBV 20, HCV 44, EtOH 13	CPC: A 61, A^+39^ (score ≥ 6)

2017	Bruix et al.	573	RCT	FACT-G EQ-5D	FACT-Hep	PR: 70.0% RR: 58.3%	II	Median (IQR) Regorafenib: 64 (54–71) Placebo: 62 (55-68)	Regorafenib: 88%M Placebo: 88%M	Spain	Regorafenib: HBV 38, EtOH 24, HCV 21, UNK 17, NASH 7, other 7 Placebo: HBV 38, EtOH 28, HCV 21, UNK 16, NASH 7, other 5	Regorafenib CPC: A 98, B 1 BCLC: A <1, B 14, C 86 Placebo CPC: A 97, B 3 BCLC: A 0, B 11, C 89

2017	Chau et al.	565	RCT	EQ-5D	FHSI-8	PR: NR RR: 63.9%	II	ITT ramucirumab: 64 (28–87) ITT placebo: 62 (25–85) AFP ≥ 400 ramucirumab: 61 (34–84) AFP ≥ 400 Placebo: 60 (25–83	Total: 84.6%M ITT ramucirumab: 83.4%M ITT placebo: 85.8%M AFP ≥ 400 ramucirumab: 77.3%M AFP ≥ 400 placebo: 84.0%M	United Kingdom	ITT ramucirumab: HBV 35.3, HCV 27.2, other 37.5 ITT placebo: HBV 35.8, HCV 27.3, other 36.9 AFP ≥400 ramicirumab: HBV 44.5, HCV 29.4, other 38.7 Placebo: HBV 50.4, HCV 21.4, other 37.4	ITT ramucirumab: CPC: A 97.9 BCLC: B 11.7, C 88.3 ITT placebo: CPC: A 97.9 BCLC: B 12.1, C 87.9 AFP ≥400 ramicirumab: CPC: A 96.6 BCLC: B 9.2, C 90.8 Placebo: CPC: A 98.5 BCLC: B 6.9, C 93.1

2017	Meyer et al.	313	RCT	QLQ-C30	QLQ-HCC18	PR: 78.4% RR: 72.4%	II	Median (IQR) TACE+placebo: 68 (63–74) TACE+sorafenib: 65 (57–71)	TACE+placebo: 88%M TACE+sorafenib: 89%M	United Kingdom	TACE+placebo: HBV 6, HCV 7, EtOH 33, HBV+HCV 2, HBV+EtOH 2, HCV+EtOH 10, HBV+HCV+EtOH 2 Sorafenib+TACE: HBV 5, HCV 12, EtOH 34, HBV+HCV 2, HBV+EtOH 2, HCV+EtOH 8, HBV+HCV+EtOH 2	TACE+placebo: CPC: A 95, B 2 TACE+sorafenib: CPC: A 93, B 4

2018	Chow et al.	360	RCT	EQ-5D	—	PR: 72% RR: 58.6%	II	Mean ± SD TARE ITT: 59.5 ± 12.9 TARE treated: 60.9 ± 11.5 Sorafenib ITT: 57.7 ± 10.6 Sorafenib treated: 57.5 ± 10.6	TARE ITT: 80.8%M TARE treated: 82.3%M Sorafenib ITT: 84.8%M Sorafenib treated: 85.2%M	Singapore	NR	TARE ITT: CPC: A 90.7, B 7.7 BCLC: A 0, B 51.1, C 48.4 TARE treated: CPC: A 90.0, B 7.7 BCLC: A 0, B 60.8, C 38.5 Sorafenib ITT: CPC: A 89.9, B 9.0 BCLC: A 0.6, B 54.5, C 44.9 Sorafenib treated: CPC: A 90.1, B 9.3 BCLC: A 0.6, B 54.3, C 45.1

2019	Eilard et al.	12	P	QLQ-C30	QLQ-HCC18	PR: 85.7% RR: 100%	III	Median (range) 55 (32–68)	75%M	Sweden	HBV 17, HCV 67, EtOH 42	CPC: A 92, B 8

2020	Baldan Ferarri et al.	16	P	QLQ-C30	QLQ-HCC18	PR: 27.1% RR: 18.6%	III	Mean ± SD 61.4 ± 9.1	100%M	Brazil	NR	CPC: A 54.6, B 45.4 BCLC: B 27.2, C 72.8

2020	Finn et al.	501	RCT	QLQ-C30	—	PR: NR RR: NR	II	Median (IQR) Atezo/Bev: 64 (56–71) Sorafenib: 66 (59–71)	Atezo/bev: 82%M Sorafenib: 83%M	United States	Atezo/bev: HBV 49, HCV 21, Nonviral 30 Sorafenib: HBV 46, HCV 22, Nonviral 32	Atezo/bev: CPC: A5 72, A6 28 BCLC: A2, B 15, C. 82 Sorafenib: CPC: A5 73, A6 27 BCLC: A 4, B 16, C 81

2020	Muszbek et al.	NR	R	QLQ-C30	—	PR: NR RR: NR	III	NR	NR	United Kingdom	NR	NR

2020	Zhu et al.	542	RCT	EQ-5D	FHSI-8	PR: NR RR: NR	II	Median Pooled Ramucirumab: 64 Placebo: 62 REACH AFP ≥400 Ramucirumab: 62 Placebo: 59 REACH-2 Ramucirumab: 64 Placebo: 64	Pooled Ramucirumab: 77.8%M Placebo: 83.6%M REACH AFP ≥400 Ramucirumab: 77.3%M Placebo: 84.0%M REACH-2 Ramucirumab: 78.2%M Placebo: 83.2%M	United States	Pooled Ramucirumab: HBV 39.2, HCV 26.3, EtOH 22.5 Placebo: HBV 45.1, HCV 24.8, EtOH 19.0 REACH AFP ≥400 Ramucirumab: HBV 44.5, HCV 29.4, EtOH 19.3 Placebo: HBV 50.4, HCV 21.4, EtOH 16.8 REACH-2 Ramucirumab: HBV 36.0, HCV 24.4, EtOH 24.4 Placebo: HBV 37.9, HCV 29.5, EtOH 22.1	Pooled Ramucirumab: CPC: A5 60.1 BCLC: B 14.2 Placebo: CPC: A5 59.7 BCLC: B 12.8 REACH AFP ≥400 Ramucirumab: CPC: A5 56.3 BCLC: B 9.2 Placebo: CPC: A5 61.8 BCLC: B 6.9 REACH-2 Ramucirumab: CPC: A5 62.4 BCLC: B 17.3 Placebo: CPC: A5 56.8 BCLC: B 21.1

2021	Galle et al.	501	RCT	QLQ-C30	QLQ-HCC18	PR: 69.1% RR: 62.6%	II	Aged ≥ 18	NR	Germany	NR	NR

2021	Kudo et al.	49	RCT	EQ-5D-3L	FACT-Hep	PR: NR RR: NR	II	Median (range) Sorafenib naïve: 68 (40–77) Sorafenib: 66.5 (47–78) All: 67 (40–78)	NR	Japan	Sorafenib naïve: HBV 20, HCV 32, Nonviral 48 Sorafenib: HBV 13, HCV 54, Nonviral 33 All: HBV 16 HCV, 43, Nonviral 41	Sorafenib naïve: CPC: 6 4, 7 68, 8 28 ALBI: I 0, II 92, III 8 BCLC: A 4, B 12, C 76, D 8 Sorafenib: CPC: 6 0, 7 83, 8 17 ALBI: I 0, II 88, III 13 BCLC: A 4, B 21, C 71, D 4 All CPC: 6 2, 7 76, 8 22 ALBI: I 0, II 90, III 10 BCLC: A 4, B 16, C 73, D 6

2021	Pereira et al.	285	RCT	QLQ-C30	—	PR: 81.3% RR: 61.0%	II	Median (IQR) TARE 67 (61–72) Sorafenib: 64 (57–71)	TARE: 92%M Sorafenib: 91%M	France	TARE: HBV 3.3, HCV 18.0, EtOH 66.4, NASH 22.1 Sorafenib: HBV 4.9, HCV 21.5, EtOH 59.5, NASH 26.4	TARE: Child–Pugh: A 86.1, B 83.1, BCLC: A 4.1, B 31.1, C 64.8 Sorafenib: Child–Pugh: A 90.8, B 9.2 C 4.3, BCLC: A 26.4, B 69.3, C 42.3

2021	Ryoo et al.	398	RCT	QLQ-C30	QLQ-HCC18	PR: NR RR: 68.1	II	NR	NR	South Korea	NR	NR

2021	Vogel et al.	954	RCT	QLQ-C30 EQ-5D	QLQ-HCC18	PR: NR RR: NR	II	Median (range) Sorafenib: 62 (22–88) Lenvatinib: 63 (20–88) Total: 62 (20–88)	Sorafenib: 84.6%M Lenvatinib 84.0%M Total: 84.3%M	Germany	NR	NR

2021	Woei-A-Jin et al.	24	P	QLQ-C30	QLQ-HCC18	PR: NR RR: 100%	III	Median (range) 62.5 (46–81)	91.7%M	The Netherlands	Cirrhosis 75.0 EtOH 70.8 HBV 25.0 HCV 20.8	CPC: A 100 BCLC: 0 12.5, A 41.6, B 45.8

2022	Abou Alfa et al.	1171	RCT	QLQ-C30	—	PR: NR RR: NR	II	Median (range) STRIDE: 65.0 (22–86) Durvalumab: 64.0 (2–-86) Sorafenib: 64 (18–88)	STRIDE: 83.2%M Durvalumab: 83.0%M Sorafenib: 86.6%M	United States	STRIDE: HBV 31.0, HCV 28.0, Nonviral 41.0 Durvalumab: HBV 30.6, HCV 27.5, Nonviral 41.9 Sorafenib: HBV 30.6, HCV 26.7, Nonviral 42.7	STRIDE: CPC: A5 75.1, A6 23.4, B7 1.0 BCLC: B 19.6, C 80.4 Durvalumab: CPC: A5 73.0, A6 24.7, B7 2.1 BCLC: B 20.6, C 79.4 Sorafenib: CPC: A5 71.2, A6 26.2, B7 2.6 BCLC: B 17.0, C 83.0

2022	Agirrezabal et al.	960	R	QLQ-C30	—	PR: NR RR: 61.2%.	III	Median (IQR) SARAH TARE: 66 (60–72) Sorafenib: 65 (58–73) IMbrave150 Atezo/Bev: (64 (56–71) Sorafenib: 66 (59–71)	SARAH 90.2%M IMbrave150 72.7%M	Germany	SARAH TARE: HBV 5, HCV 23, EtOH 62, NASH 21, sorafenib: HBV 7, HCV 22, EtOH 56, NASH 27 IMbrave150 Atezo/bev: HBV 49, HCV 21, sorafenib: HBV 46, HCV 22, NV 32	SARAH TARE: CPC: A 83, B 16 BCLC: A 4, B 28, C 68 Sorafenib: CPC: A 84, B 16 CPC B 0% UNK BCLC: A 5, B 27, C 67 IMbrave150 Atezoliozumab/bevacizumab: CPC: A 99, B <1 BCLC: A 2, B 15, C 82 Sorafenib: CPC: A 100, 0 B BCLC: A 4, B 16, C 81

2022	Charonpongsuntorn et al.	30	P	QLQ-C30	QLQ-HCC18	PR: NR RR: 100%	III	Median 58.06	90%M	Thailand	HBV 63.3, HCV 10, EtOH 16.7, NASH 10	CPC: A 100

2022	Freemantle et al.	707	RCT	EQ-5D-5L	—	PR:NR RR: NR	II	NR	NR	United States	NR	NR

2022	Qiao et al.	15	P	QLQ-C30	—	PR: 50% RR: 41.6%	III	Median (IQR) TACE+anti-PD-1: 59 (53–63) TACE: 60 (54–64)	TACE+anti-PD-1: 100%M TACE: 100%M	China	TACE+anti-PD-1: HBV 86.7, HCV 0, EtOH 13.3 TACE: HBV 86.7, HCV 13.3, EtOH 0	TACE+anti-PD-1 CPC: A 80, B 20 CNLC: Ia 33.3, Ib 46.7, Ia/IIb 20 TACE CPC: A 80, B 20 CNLC Ia 20, Ib 80, Ia/IIb 0

2022	Yau et al.	743	RCT	EQ-5D-3L	FACT-Hep	PR: 56.2% RR: 4.4%	II	Median (IQR) Nivolumab: 65 (57–71) Sorafenib: 65 (58–72)	Nivolumab: 85%M Sorafenib: 85%M	Hong Kong	Nivolumab: HBV 31, HCV 23, Nonviral 45 Sorafenib: HBV 31, HCV 23, Nonviral 45	Nivolumab: CPC: A 98 BCLC: A 4, B 14, C 82 Sorafenib: CPC: A 96 BCLC: A 5, B 17, C 78

2022	Zou et al.	46	P	QLQ-C30	QLQ-HCC18	PR: NR RR: 100%	III	Median (range) 58 (45–69)	89%M	China	HBV 93.4, HCV 2.2, HBV+HCV 2.2, other 2.2	CPC: 5 52.2, 6 47.8, 7 0 BCLC: B 26.1, C 73.9

Abbreviations: AFP, alpha fetoprotein; ALBI, albumin-bilirubin score; anti-PD-1, programmed cell death protein 1 inhibitor; Atezo/bev, atezolizumab/bevacizumab; BCLCS, Barcelona Clinic Liver Cancer Score; CNLC, China liver cancer staging; CPC, Child–Pugh class; CRYP, cryptogenic; EC, exclusion criteria; EQ-5D, EuroQol-5 dimension questionnaire; EQ-5D-3L, EuroQol-5 dimension questionnaire–three level; EQ-5D-5L, EuroQol-5 dimension questionnaire–five level; EtOH, ethanol; F, female; FACT, Functional Assessment of Cancer Therapy; FACT-G, Functional Assessment of Cancer Therapy–General Questionnaire; FACT-Hep, Functional Assessment of Cancer Therapy–Hepatobiliary Questionnaire; FACT-HS, Functional Assessment of Cancer Therapy–Hepatobiliary Subscale; FHSI-8, Functional Assessment of Cancer Therapy Hepatobiliary Cancer Symptom Index-8; HBV, hepatitis B virus; HCV, hepatitis C virus; HRQOL, health-related quality of life; IQR, interquartile range; ITT, intention to treat; M, male; NASH, nonalcoholic steatohepatitis; NR, not reported; P, prospective cohort study; PR, participation rate; QLQ-C30, Quality of life Questionnaire Core 30; QLQ-HCC18, Quality of Life Questionnaire Hepatocellular Carcinoma 18; R, retrospective cohort study; RCT, randomised controlled trial; RR, response rate; SARAH, sorafenib versus radioembolisation in advanced hepatocellular carcinoma; SD, standard deviation; SF-36, Medical Outcomes Survey Short-Form 36; STRIDE, single tremelimumab regular interval durvalumab; TACE, transarterial chemoembolisation; TARE, transarterial radioembolisation; UNK, unknown.

**Table 2:** Key findings of included studies.

**Year**	**Author**	**Method of follow-up**	**Follow-up interval**	**Comparison groups**		
				**Baseline/placebo**	**Systemic therapies**	**Systemic therapy vs. TARE**
2009	Cheng et al.	Self-administered questionnaires (FACT-Hep and FHSI-8)	Baseline and Week 12 of treatment	Yes	Sorafenib	No
		Both treatment groups had similar total scores on the FHSI-8 questionnaire, and scores with the FACT-Hep questionnaire showed no difference in quality of life between the two groups (data not shown).				

2012	Brunocilla et al.	Self-administered questionnaires (FACT-Hep, FHSI-8, and FACT-G)	Baseline, 1 week, 1 month, and 2 months post-treatment	Yes	Sorafenib	No
		All domains and total scores except for social/family well-being and emotional well-being saw a significant worsening (*p* ≤ 0.02) within the first week of treatment. Physical well-being showed a significant worsening both 1- and 2-months post-treatment (*p* = 0.01) while hepatobiliary subscale only significantly worsened 2 months post-treatment (*p* = 0.01). In terms of total scores, they were only significantly reduced after the first week of treatment, with 1 and 2 months showing nonsignificant worsening. The emotional well-being domain displayed a significant improvement 1- and 2-months post-treatment (*p* = 0.02 and *p* = 0.01, respectively).				

2013	Johnson et al.	Self-administered questionnaire (QLQ-C30)	Baseline, every 6 weeks, and end of treatment	Yes	Brivanib vs. sorafenib	No
		A statistically significant worsening in the HRQOL scores of both sorafenib and brivanib groups was observed. The decline was to a greater extent in the brivanib patients; however, statistical significance between the two treatments was not reported.				

2013	Montella et al.	Self-administered questionnaires (FHSI-8)	Before treatment and 2 and 4 months post-treatment	Yes	Sorafenib	No
		There was no significant change in HRQOL reported after commencing treatment with sorafenib.				

2014	Chow et al.	Self-administered questionnaires (EQ-5D)	Baseline, every month during treatment, and 6-month intervals thereafter	Yes	Sorafenib	No
		Overall, patients' EQ-5D score increased over time (*β* = 0.374). Patients with BCLC Stage B decreased over time (*β* = −0.004), while patients with BCLC Stage C increased over time (*β* = 0.001)				

2016	Koeberle et al.	Self-administered questionnaires (FACT-HS)	Baseline and every 2 weeks for 12 weeks	Yes	Sorafenib vs. sorafenib+everolimus	No
		The FACT-HS score was similar over time in both treatment groups. The odds of having a clinically relevant improvement in the FACT-HS score (a change of ≥ 5 points) was higher in patients in arm S compared with those in arm S+E (OR with 95% CI 3.2 (1.0–10.9); *p* = 0.03). Significant differences in change from baseline for physical well-being (*p* = 0.02) in favour of the sorafenib group and mood (*p* = 0.02) in favour of the sorafenib+everolimus group. Patients in the sorafenib+everolimus group reported a greater worsening until 12 weeks.				

2016	Shomura et al.	Self-administered questionnaires (SF-36)	Baseline and every 3 months post-treatment	No	Sorafenib	No
		13 (24%) patients who were able to take sorafenib for at least 1 year did not see any significant decline in their HRQOL scores and reported above 40 points in all domains. All HRQOL domains reported scores at baseline below the national average of 50 except for vitality, social functioning, and mental health. All scores ended below 50 points at the time of imminent death. A significant worsening (*p* ≤ 0.032) at every time interval in relation to imminent death was reported for the domains physical functioning, role physical, and vitality. The domains bodily pain, social functioning, and role emotional saw no significant worsening across any time interval. Median overall survival (median [IQR]) was 9.6 (0.8–16.3) months, and previous curative therapy (*p* < 0.001) as well as a physical functioning score of ≥ 40 at baseline (*p* = 0.031) was associated with longer overall survival upon multivariate analysis. Upon multivariate analysis of baseline scores associated with treatment duration, only social functioning (*p* = 0.049) was associated with longer survival. The domains bodily pain, general health, social functioning, and mental health did not show significant changes across the time measured. Domains relating to physicality such as physical functioning, role physical, and vitality saw significant decline nearing death.				

2017	Bruix et al.	Self-administered questionnaires (FACT-Hep+FACT-G+EQ-5D)	Baseline and end of trial	Yes	Regorafenib	No
		No clinically meaningful differences were noted between the regorafenib and placebo groups in HRQOL. Overall changes from baseline in EQ-5D and FACT-Hep were similar in the two groups. In the least mean squares time adjusted analysis of EQ-5D and Fact-Hep, the scores were lower in the regorafenib group than the placebo group (*p* < 0.001), but the minimally important thresholds for the differences were not met.				

2017	Chau et al.	Self-administered questionnaires (FHSI-8+EQ-5D)	Baseline, 6 weeks, and then every 12 weeks thereafter	Yes	Ramucirumab	No
		Upon comparison of the ITT group who received ramucirumab and placebo, no significant difference was found (*p* = 0.3722); however, when comparing patients who had AFP ≥ 400 ng/mL, the ramucirumab group had a significantly (*p* = 0.0381) smaller deterioration in FHSI-8 total score. Upon analysis of change in tumour size and FHSI-8 scores, there was a linear relationship in both ITT (slope = 0.1312, *p* = 0.0023) and AFP ≥ 400 ng/mL (slope = 0.1979, *p* = 0.1979) patients showing that more tumour progression may lead to a higher deterioration in HRQOL. In the ITT population, there was no significant difference in the time to initial deterioration in FHSI-8 score between treatment and placebo (HR 95% CI, *p* value) (1.037 [0.802–1.341], *p* = 0.782). In AFP ≥ 400 ng/mL patients, the time to first deterioration approached significance (HR 95% CI, *p* value) (0.690 [0.470–1.014], *p* = 0.054). *p* values for EQ-5D results were not reported, but there is minimal difference in ramucirumab vs. placebo for both patient groups.				

2017	Meyer et al.	Self-administered questionnaire (QLQ-C30 and QLQ-HCC18)	Baseline, Week 10, and every 6 weeks thereafter. Median follow-up time was 620.0 days (95% CI 572–784)	Yes	Sorafenib	Sorafenib+TACE vs. placebo+TACE
		After 360 days, the QLQ-C30 domains social (*p* = 0.045) and role functioning (*p* = 0.05) had significantly worsened by up to 6% in the sorafenib group. Diarrhoea (*p* = 0.0095) and appetite loss (*p* = 0.0018) domains also significantly worsened by up to 13% and 10%, respectively. Using QLQ-HCC18, the nutritional problem domain worsened by up to 7% (*p* = 0.0084) in the sorafenib group. Notably, the sorafenib group encountered well documented adverse effects such as stomatitis, palmar-plantar erythrodysesthesia, and diarrhoea, which could have led to worse HRQOL outcomes.				

2018	Chow et al.	Self-administered questionnaire (EQ-5D)	Baseline, every month during treatment, and 6-month intervals thereafter	No	Sorafenib	Sorafenib vs. TARE
		Interaction between treatment (TARE vs. sorafenib) and time: treatment (*p* value), time (*p* value), and treatment × time (*p* value) (where time is in months). There were no statistically significant differences found between the group who received TARE and the group who received sorafenib in either population.				

2019	Eilard et al.	Self-administered questionnaires (QLQ-C30 and QLQ-HCC18)	Baseline, 1 and 4 weeks, and every 4 weeks thereafter	No	Sorafenib	No
		A significant worsening in the QLQ-C30 domains: nausea and vomiting (*p* = 0.043), appetite loss (*p* = 0.008), and pain (*p* = 0.045) was observed. A similar but nonsignificant worsening was seen in the domains social functioning, cognitive functioning, physical functioning, role functioning, and QOL. No significant changes to QLQ-HCC18 domains were seen, but a similar nonsignificant worsening was observed in the domains fatigue and fever and pain. The nutrition domain saw the highest symptom burden at 4 weeks post-treatment, while the diarrhoea domain saw the highest symptom burden at 8 weeks post-treatment.				

2020	Baldan Ferrari et al.	Self-administered questionnaires (QLQ-C30 and QLQ-HCC18)	Baseline, 1 and 4 weeks, and every 4 weeks thereafter	No	Sorafenib	No
		A significant worsening in the QLQ-C30 domains emotional functioning (*p* = 0.0313) and pain (*p* = 0.0313) was found after the first treatment cycle. Patients who reported pain had a statistically significant worsening in the QLQ-HCC18 pain domain (*p* = 0.0449) and patients who presented with palmar-plantar erythrodysesthesia had a significant worsening in the body image domain (*p* = 0.0442).				

2020	Finn et al.	Self-administered questionnaires (QLQ-C30)	Baseline, Day 1 of each treatment cycle, and every 3 months for 1 year after treatment cessation	No	Atezolizumab/bevacizumab vs. sorafenib	No
		Treatment with atezolizumab/bevacizumab delayed time to deterioration compared to sorafenib.				

2020	Muszbek et al.	Self-administered questionnaire (QLQ-C30)	NR	No	Sorafenib	Sorafenib vs. TACE
		Patients who received radiation therapy in the SARAH trial had significantly improved global health status in the QLQ-C30 questionnaire (*p* = 0.0048) when compared to the sorafenib group. Radiation therapy was found to cost less than sorafenib (£29,530 vs. £30,957) yet result in higher total QALYs (1.982 vs. 1.381). This benefit was mostly because of progression free survival.				

2020	Zhu et al.	Self-administered questionnaires (FHSI-8 and EQ-5D)	Baseline, 6 weeks following first treatment, and every 12 weeks thereafter in REACH. Baseline and every 6 weeks in REACH-2	Yes	Ramucirumab	No
		In the pooled population with AFP ≥ 400 ng/mL, the median time to deterioration in FHSI-8 total score was prolonged with ramucirumab compared to placebo (3.3 vs. 1.9 months; HR 0.725; (95% CI 0.559–0.941), *p* = 0.0152). There was no significant difference in time to deterioration of EQ-5D score between ramucirumab and placebo groups (*p* = 0.24).				

2021	Galle et al.	Self-administered questionnaires (QLQ-C30 and QLQ-HCC18)	Baseline and every 3 months for 1 year after treatment. After treatment follow-up time: median (IQR) 8.6 months (6.2–10.8)	No	Atezolizumab/bevacizumab vs. sorafenib	No
		Atezolizumab/bevacizumab was associated with a significant reduced risk of deterioration in the QLQ-C30 domains (HR [95% CI]): fatigue (0.61 [0.46–0.81]), pain (0.46 [0.34–0.62]), appetite loss (0.57 [0.40–0.81]), diarrhoea (0.23 [0.16–0.34]), nausea and vomiting (0.39 [0.26–0.60]), dyspnoea (0.54 [0.37–0.79]), insomnia (0.67 [0.46–0.99]), emotional functioning (0.47 [0.31–0.71]), social functioning (0.71 [0.51–0.98]), and cognitive functioning (0.56 [0.40–0.79]). QLQ-HCC18 domains that saw a significant reduced risk of deterioration were (HR [95% CI]) fatigue (0.60 [0.45–0.80]), pain (0.65 [0.46–0.92]), abdominal swelling (0.57 [0.37–0.86]), body image (0.71 [0.52–0.97]), and nutrition (0.56 [0.40–0.79]). Although the significance of the change from baseline is not reported, the reduction in HRQOL scores is considerably less when comparing atezolizumab/bevacizumab vs. sorafenib.				

2021	Kudo et al.	Self-administered questionnaires (FACT-Hep and EQ-5D-3L)	Baseline and the first day of every cycle thereafter. Each cycle was 2 weeks apart.	Yes	Nivolumab	No
		Mixed model with repeated measures analyses were conducted for EQ-5D-3L and FACT-Hep. EQ-5D visual analogue scale score (LS −3.2, 95% CI [−8.5 to 2.0]) remained stable over time with no meaningful decline observed through week 36. The overall utility index was similar, with no clinically meaningful decline observed through week 28 (LS −0.063, 95% CI [−0.118 to −0.007]). FACT-Hep also showed similar results with no clinically meaningful decline observed in 91.7% of evaluable timepoints. The LS means for FACT-Hep and HCS were −7.9 and −3.6 months, respectively.				

2021	Pereira et al.	Self-administered questionnaires (QLQ-C30)	Baseline, 1 and 3 months post-treatment, and every 3 months thereafter. After treatment: median (IQR) (months) TARE: 29.2 (21.9–34.8) Sorafenib: 29.6 (21.4–35.1)	Yes	Sorafenib	Sorafenib vs. TARE
		There was a significant treatment effect for the domains: fatigue (*p* < 0.0001), pain (*p* = 0.030), appetite loss (*p* < 0.0001), diarrhoea (*p* < 0.0001), nausea and vomiting (*p* = 0.05), emotional functioning (*p* < 0.001), social functioning (*p* < 0.0001), role functioning (*p* = 0.003), and QOL (*p* = 0.006). Of these, the only domains to show a significant treatment by time interaction were diarrhoea (*p* < 0.0001), nausea and vomiting (*p* = 0.07), and social functioning (*p* = 0.003). Upon analysis of the QOL responses, there was a statistically significant worse responses (TARE % vs. Sorafenib % (*p* value)) in the domains: nausea and vomiting (47.2% vs. 33.6% [*p* = 0.012]), appetite loss (69.1% vs. 41.0% [*p* < 0.0001]), diarrhoea (70.8% vs. 26.4% [*p* < 0.0001]), and social functioning (66.5% vs. 46.7% [*p* = 0.004]). TARE treatment was associated with a significant reduced risk in deterioration in the domains social functioning, physical functioning, role functioning, and QOL. Although the other domains were not significant, the time to deterioration on sorafenib was still shorter. Overall, TARE treatment displayed a reduced risk in time to deterioration of QOL compared to sorafenib.				

2021	Ryoo et al.	Self-administered questionnaire (QLQ-C30 and QLQ-HCC18)	Baseline at Weeks 2, 3, 4, 6, 9, 12, and 18 and then every 9 weeks until 1 year or end of treatment	Yes	Pembrolizumab	No
		LS mean change from baseline to Week 12 in QLQ-HCC18 was similar between pembrolizumab and placebo groups. The proportion of patients reporting improvement, stable, or deterioration in QOL was generally similar between pembrolizumab and placebo groups. The change from baseline to Week 12 in QLQ-C30 were similar in both functional and symptom domain scores between pembrolizumab and placebo arms. Time to deterioration was not significantly different between the pembrolizumab and placebo groups. Generally, there was no significant difference for HRQOL scores between pembrolizumab and placebo groups.				

2021	Vogel et al.	Self-administered questionnaire (QLQ-C30 and QLQ-HCC18)	Baseline and Day 1 of each postbaseline cycle and end of treatment. Each cycle was 28 days	Yes	Sorafenib vs. lenvatinib	No
		Upon analysis, patients treated with lenvatinib saw significant improvements in time to deterioration (HR [95% CI]), in the domains fatigue (0.83 [0.69–0.99]), pain, (0.80 [0.66–0.96]), diarrhoea (0.52 [0.42–0.65]), body image (0.81 [0.67–0.98]), nutrition (0.74 [0.60–0.90]), and the EQ-VAS score (0.83 [0.69–0.99]). No other significant differences in time to deterioration was reported. There were no significant differences in individual domain scores, although the LS mean score for diarrhoea was numerically significant in favour of Lenvatinib. The LS mean score for constipation favoured sorafenib. There were no significant differences in global health scores, EQ-VAS score, or the EQ-5D index.				

2021	Woei-A-Jin et al.	Self-administered questionnaire (QLQ-C30 and QLQ-HCC18)	Baseline, Day 12, Day 26, and follow-up post-treatment.	Yes	Dovitinib	No
		HRQOL generally decreased during dovitinib treatment. Fatigue and social functioning worsened on Day 12 compared to baseline (*p* < 0.023) but showed recovery at Day 26 and follow-up. Global health score had deteriorated significantly (*p* < 0.006) at follow-up. Cognitive and emotional functioning scales were not affected by the treatment.				

2022	Abou Alfa et al.	Self-administered questionnaires (QLQ-C30)	Baseline and every 4 weeks thereafter	Yes	STRIDE vs. durvalumab vs. sorafenib	No
		There was prolonged time to deterioration of quality of life for the durvalumab group compared to the sorafenib group.				

2022	Agirrezabal et al.	Self-administered questionnaires (QLQ-C30)	Baseline, 1 and 4 weeks, and every 4 weeks thereafter.	No	Atezolizumab/bevacizumab vs. sorafenib	Atezolizumab/bevacizumab vs. TARE Sorafenib vs. TARE
		Time to deterioration in the SARAH trial was significantly (HR, 95% CI, [*p* value]) (0.69, 0.54–0.88, [*p* = 0.003]) longer for TARE (6.93 months) vs. sorafenib (4.30 months). Upon base case analysis, the atezolizumab/bevacizumab had the longest median TTD (11.23 months), while TARE (8.64 months) was second and sorafenib in both SARAH and IMbrave150 trials showed the lowest TTD (5.52 and 3.58 months, respectively). The difference between TARE and atezolizumab/bevacizumab was not statistically significant (HR, 95% CI, [*p* value]) (1.06, 0.75–1.50 [*p* = 0.725]). Upon sensitivity analysis, TARE had the highest median TTD (19.88 months), and there was no statistical difference again between TARE and atezolizumab/bevacizumab (HR, 95% CI, [*p* value]) (0.66, 0.36–1.19 [*p* = 0.163]). Sorafenib had the shortest TTD in HRQOL, with statistically significant differences in both base case and sensitivity analyses.				

2022	Charonpongsuntorn et al.	Self-administered questionnaires (QLQ-C30 and QLQ-HCC18)	Baseline and every 12 weeks thereafter	Yes	Atezolizumab/bevacizumab	No
		The coefficients at baseline and 3 months were not significantly different (difference, 95% CI) (−5.58, −16.44 to 5.27). The coefficients at 6 and 9 months were significantly lower when compared to baseline (difference, 95% CI) (−17.82, −29.8 to −5.84) and (−33.46, −47.83 to −19.06), respectively. The *p* value was not reported. The physical function scores were not significantly different from baseline to 3 months (difference, 95% CI) (−5.96, −15.27 to 3.34) but did significantly decline at 6 and 9 months compared to baseline (difference, 95% CI) (−12.58, −22.87 to 2.29) and (−23.76, −36.18 to −11.40), respectively. The *p* value was not reported. The significance of the steady increase in QLQ-HCC18 score was not reported.				

2022	Freemantle et al.	Self-administered questionnaires (EQ-5D-5L)	Baseline, every 4 weeks until Week 25, and then 8 weeks thereafter	Yes	Cabozantinib	No
		At baseline, mean EQ-5D-5L scores were higher for the cabozantinib group compared to the placebo group. There were statistically significant differences in favour of the placebo group until Week 33. There were statistically significant differences in favour of the placebo group until Week 21. At the end of follow-up, the mean EQ-5D-5L score was significantly higher for the cabozantinib group for the domains mobility (*p* < 0.0001), self-care (*p* < 0.0033), usual activities (*p* < 0.0001), and pain/discomfort (*p* < 0.0005).				

2022	Qiao et al.	Self-administered questionnaires (QLQ-C30)	Baseline and every 3 months	Yes	Pembrolizumab	No
		All patients completed quality of life assessments, and none of them had experienced a deterioration in quality of life in either group.				

2022	Yau et al.	Self-administered questionnaires (FACT-Hep and EQ-5D-3L)	Baseline and every cycle thereafter	Yes	Nivolumab vs. sorafenib	No
		Mean baseline FACT-Hep scores were similar in both treatment groups. Adjusted analyses yielded minimally important differences in least squares means favouring nivolumab. No subscales favoured sorafenib. Time to treatment deterioration was substantially delayed with nivolumab. Nivolumab showed improved scores and lengthened time to deterioration compared to sorafenib.				

2022	Zou et al.	Self-administered questionnaires (EORTC QLQ-C30 and QLQ-HCC18)	Baseline and after treatment	Yes	Lenvatinib vs. lenvatinib+PD-1 inhibitor (pembrolizumab or toripalimab)	No
		There were significant improvements after anti-PD-1 therapy compared to monotherapy for the emotional function (*p* = 0.01), global HRQOL (*p* = 0.00), and diarrhoea (*p* = 0.01) subscales. There were significant improvements after anti-PD-1 therapy compared to monotherapy for the pain (*p* = 0.01) and fatigue (*p* = 0.03) subscales. Emotional functioning and overall HRQOL were improved significantly in patients with HCC after initiation of treatment with anti-PD-1 antibodies plus lenvatinib. The addition of anti-PD-1 therapy reduced the incidence of deterioration on the QLQ-HCC18 symptom scales when compared to lenvatinib monotherapy.				

Abbreviations: AFP, alpha fetoprotein; AUC, area under the curve; BSC, best supportive care; CI, confidence interval; EQ-5D, EuroQol-5 dimension questionnaire; EQ-5D-3L, EuroQol-5 dimension questionnaire–three level; EQ-5D-5L, EuroQol-5 dimension questionnaire–five level; EQ-VAS, EuroQol-visual analogue scales; FACT, Functional Assessment of Cancer Therapy; FACT-G, Functional Assessment of Cancer Therapy–General Questionnaire; FACT-Hep, Functional Assessment of Cancer Therapy–Hepatobiliary Questionnaire; FHSI-8, Functional Assessment of Cancer Therapy Hepatobiliary Cancer Symptom Index-8; HCS, hepatobiliary cancer subscale; HR, hazard ratio; HRQOL, health-related quality of life; IQR, interquartile range; ITT, intention to treat; KW, Kruskal–Wallis test; LS, least squares; MWU, Mann–Whitney *U* test; NE, not evaluated; NR, not reported; OR, odds ratio; PD-1, programmed cell death protein 1; QALY, quality-adjusted life-year; QLQ-C30, Quality of Life Questionnaire Core 30; QLQ-HCC18, Quality of Life Questionnaire Hepatocellular Carcinoma 18; QOL, quality of life; REACH, ramucirumab versus placebo as second-line treatment in patients with advanced hepatocellular carcinoma following first-line therapy with sorafenib; SARAH, sorafenib versus radioembolisation in advanced hepatocellular carcinoma; SD, standard deviation; SF-36, Medical Outcomes Survey Short-Form 36; STRIDE, single tremelimumab regular interval durvalumab; TACE, transcatheter chemoembolisation; TARE, transcatheter radioembolisation; TTD, time to deterioration; VAS, visual analogue scale; WTS, Wilcoxon two sample test.

## Data Availability

The data that supports the findings of this study are available in the supporting information of this article.

## Nomenclature

atezo/bev: atezolizumab/bevacizumab
BCLC: Barcelona Clinic Liver Cancer
EQ-5D: EuroQol 5D
FACT-G: Functional Assessment of Cancer Therapy–General Questionnaire
FACT-Hep: Functional Assessment of Cancer Therapy–Hepatobiliary Questionnaire
FACT-HS: Functional Assessment of Cancer Therapy–Hepatobiliary Subscale
FHSI-8: Functional Assessment of Cancer Therapy Hepatobiliary Cancer Symptom Index
HCC: hepatocellular carcinoma
HRQOL: health-related quality of life
MeSH: Medical Subject Headings
NHMRC: National Health and Medical Research Council
PD-1: programmed cell death protein 1
PRISMA: preferred reporting items for systematic reviews and meta-analyses
PROSPERO: International prospective register of systematic reviews
PRS: post-radioembolisation syndrome
QLQ-C30: Quality of Life Questionnaire Core 30
QLQ-HCC18: Quality of Life Questionnaire Hepatocellular Carcinoma
QOL: quality of life
RCT: randomised-control trials
RoB 2: revised Cochrane risk-of-bias tool for randomised trials
ROBINS-I: risk of bias in nonrandomised studies of interventions
SF-36: Medical Outcomes Survey Short-Form 36
STRIDE: single tremelimumab regular interval durvalumab
TACE: transcatheter chemoembolisation
TARE: transarterial radioembolisation
TTD: time to deterioration

## Supporting Information

Supporting Information 1 Table S1: PRISMA 2020 checklist for systematic reviews.

Supporting Information 2 Table S2: PRISMA 2020 checklist for abstracts of systematic reviews.

Supporting Information 3 Table S3: Searching inputs used for PubMed and EMBASE for the systematic literature search.

Supporting Information 4 Table S4: Complete results of included studies.

Supporting Information 5 Table S5: Canada Institute of Health Economics (IHE) quality appraisal checklist for included case series.
