# Supplementary material for: Hepatocellular Carcinoma and Health-Related Quality of Life: A Systematic Review of Outcomes From Systemic Therapies
Source: Int J Hepatol. 2025 Apr 7;2025:1083642. doi: 10.1155/ijh/1083642 (PMC11996279; doi:10.1155/ijh/1083642)
Supplement: Supporting Information 3 — Table S3: Searching inputs used for PubMed and EMBASE for the systematic literature search. [file 1083642.f3.docx]

**Table S3: Searching inputs used for PubMed and EMBASE for the systematic literature search**

| **Database** |  | **Search Terms** |
| --- | --- | --- |
| PubMed (limit from 1^st^ January 2000 to 1^st^ May 2024) | 1 | "Carcinoma, Hepatocellular"[MeSH Terms] |
|  | 2 | "quality of life"[MeSH Terms] |
|  | 3 | english[Language] |
|  | 4 | ("2000"[PDAT]:"3000"[PDAT]) |
|  | 5 | NOT "child"[MeSH Terms] |
| EMBASE (1^st^ January 2000 to 1^st^ May 2024) | 1 | carcinoma, hepatocellular.mp. or liver cell carcinoma/ |
|  | 2 | hepatoma.mp. or liver cell carcinoma/ |
|  | 3 | hepatocellular carcinoma.mp. or liver cell carcinoma/ |
|  | 4 | quality of life.mp. or "quality of life"/ |
|  | 5 | HRQOL.mp. |
|  | 6 | 1 or 2 or 3 |
|  | 7 | 4 or 5 |
|  | 8 | 6 and 7 |
|  | 9 | Limit 8 to (human and English language and (adult <18 to 64 years> or aged <65+ years>)) |
