# Supplementary material for: Hepatocellular Carcinoma and Health-Related Quality of Life: A Systematic Review of Outcomes From Systemic Therapies
Source: Int J Hepatol. 2025 Apr 7;2025:1083642. doi: 10.1155/ijh/1083642 (PMC11996279; doi:10.1155/ijh/1083642)
Supplement: Supporting Information 4 — Table S4: Complete results of included studies. [file 1083642.f4.docx]

**Table S4: Complete results of included studies**

| Year | Author | | Method of Follow-Up | | Follow-up interval | | Comparison Groups | | | | | | |  |
| --- | --- | --- | --- | --- | --- | --- | --- | --- | --- | --- | --- | --- | --- | --- |
|  |  |  |  |  |  |  | Baseline/Placebo | | Systemic Therapies | | Systemic Therapy vs TARE | |  |  |
| **2009** | **Cheng et al.** | | Self-Administered Questionnaires (FACT-Hep and FHSI-8) | | Baseline and week 12 of treatment. | | Yes | | Sorafenib | | No | |  |  |
|  |  |  | Both treatment groups had similar total scores on the FHSI-8 questionnaire and scores with the FACT–Hep questionnaire showed no difference in quality of life between the two groups (data not shown). | | | | | | | | | | |  |
| **2012** | **Brunocilla et al.** | | Self-Administered Questionnaires (FACT-Hep, FHSI-8, FACT-G) | | Baseline, 1 week, 1 month and 2 months post treatment | | Yes | | Sorafenib | | No | |  |  |
|  |  |  | Changes in HRQOL from baseline at: 1 week median (p-value), 1 month median (p-value), 2 months median (p-value)   - Physical Wellbeing: -8.33 (p < 0.01), -3.93 (p = 0.01), -3.57 (p = 0.01) - Social/Family Wellbeing: 0.00 (p = 0.99), -1.77 (p = 0.42), -3.87 (p = 0.42) - Emotional Wellbeing: 0.00 (p = 0.28), 8.33 (p = 0.02), 8.33 (p = 0.01) - Functional Wellbeing: -3.57 (p = 0.02), -3.57 (p = 0.48), -3.57 (p = 0.11) - Hepatobiliary Subscale: -4.17 (p = 0.01), -4.17 (p = 0.07), -5.56 (p = 0.01) - FACT-G: -4.63 (p = 0.02), -1.30 (p = 0.55), -1.36 (p = 0.24) - FACT-Hep: -2.22 (p = 0.01), -0.93 (p = 0.25), -3.92 (p = 0.07) - FHSI-8: -6.25 (p = 0.02), -1.56 (p = 0.33), -1.56 (p = 0.25)   All domains and total scores except for social/family wellbeing and emotional wellbeing saw a significant worsening (p ≤ 0.02) within the first week of treatment. Physical wellbeing showed a significant worsening both 1- and 2-months post treatment (p = 0.01) while hepatobiliary subscale only significantly worsened 2 months post treatment (p =0.01). In terms of total scores, they were only significantly reduced after the first week of treatment, with 1 and 2 months showing non-significant worsening. The emotional wellbeing domain displayed a significant improvement 1- and 2-months post treatment (p = 0.02 and p = 0.01 respectively). | | | | | | | | | | |  |
| **2013** | **Johnson et al.** | | Self-Administered Questionnaire (QLQ-C30) | | Baseline, every 6 weeks and end of treatment. | | Yes | | Brivanib vs Sorafenib | | No | |  |  |
|  |  |  | Change in QLQ-C30 Score: Sorafenib Mean (SD) Median (Range), Brivanib Mean (SD) Median (Range), (p-value)   - Physical Function (Baseline): 83 (17) 87 (0-100), 83 (17) 87 (0-100), (p = 0.3181) - Physical Function (at week 12): -18 (28) -7 (-100-87), -24 (29), -13 (-100-53), (p = 0.0002) - Role Function (Baseline): 84 (25) 100 (0-100), 85 (23) 100 (0-100), (p = 0.6061) - Role Function (at week 12): -20 (33) -17 (-100-83), -28 (34) -33 (-100-67), (p = 0.0002)   A statistically significant worsening in the HRQOL scores of both sorafenib and brivanib groups was observed. The decline was to a greater extent in the Brivanib patients however statistical significance between the two treatments was not reported. | | | | | | | | | |  |  |
| **2013** | **Montella et al.** | | Self-Administered Questionnaires  (FHSI-8) | | Before treatment, 2- and 4-months post treatment | | Yes | | Sorafenib | | No | |  |  |
|  |  |  | There was no significant change in HRQOL reported after commencing treatment with sorafenib. | | | | | | | | | | |  |
| **2014** | **Chow et al.** | | Self-Administered Questionnaires (EQ-5D) | | Baseline, every month during treatment and 6-month intervals thereafter | | Yes | | Sorafenib | | No | |  |  |
|  |  |  | Overall, patients’ EQ-5D score increased over time (β = 0.374). Patients with BCLC stage B decreased over time (β = -.004) while patients with BCLC stage C increased over time (β = 0.001) | | | | | | | | | | |  |
| **2016** | **Koeberle et al.** | | Self-Administered Questionnaires (FACT-HS) | | Baseline and every 2 weeks for 12 weeks. | | Yes | | Sorafenib vs Sorafenib + Everolimus | | No | |  |  |
|  |  |  | The FACT-HS score was similar over time in both treatment groups. The odds of having a clinically relevant improvement in the FACT-HS score (a change of ≥5 points) was higher in patients in arm S compared with those in arm S + E (OR with 95% CI 3.2 (1.0–10.9); p = 0.03). Significant differences in change from baseline for physical wellbeing (p = 0.02) in favour of the sorafenib group and mood (p = 0.02) in favour of the sorafenib + everolimus group. Patients in the sorafenib + everolimus group reported a greater worsening until 12 weeks. | | | | | | | | | | |  |
| **2016** | **Shomura et al.** | | Self-Administered Questionnaires  (SF-36) | | Baseline and every 3 months post treatment | | No | | Sorafenib | | No | |  |  |
|  |  |  | 13 (24%) patients who were able to take sorafenib for at least one year did not see any significant decline in their HRQOL scores and reported above 40 points in all domains.  Changes in HRQOL scores in time points prior to death: 12 months median (p-value), 9 months median (p-value), 6 months median (p-value), 3 months median (p-value), imminent death median (p-value). All (p-values) are in comparison to imminent death unless otherwise stated.   - Physical Functioning: 48.8 (p = 0.07), 50.6 (p = 0.000), 47.0 (p = 0.001), 43.4 (p = 0.001), 36.2 - Role Physical: 49.1 (p = 0.003), 42.4 (p = 0.032), 40.1 (p = 0.001), 35.8 (NR), 30.8 - Bodily Pain: 44.7, 47.0, 47.4, 44.7, 44.7, no significant differences reported - General Health: 43.7 (NR), 48.2 (p = 0.002), 44.2 (p = 0.014), 44.2 (NR), 30.8 - Vitality: 59.5 (p = 0.031 to 6 months) (p = 0.038 to 3 months) (p = 0.002), 51.4 (p = 0.000), 43.4 (p = 0.0122), 43.4 (p = 0.006), 40.2 - Social Functioning: 57.0, 50.6, 53.8, 57.0, 44.1, no significant differences reported - Role Emotional: 45.7, 47.8, 51.9, 47.8, 41.6, no significant differences reported - Mental Health: 51.8 (NR), 54.5 (p = 0.007), 49.1 (p = 0.044), 51.8 (NR), 45.1   All HRQOL domains reported scores at baseline below the national average of 50 except for vitality, social functioning, and mental health. All scores ended below 50 points at the time of imminent death. A significant worsening (p ≤ 0.032) at every time interval in relation to imminent death was reported for the domains physical functioning, role physical and vitality. The domains bodily pain, social functioning, and role emotional saw no significant worsening across any time interval. Median overall survival (median (IQR)) was 9.6 (0.8-16.3) months and previous curative therapy (p < 0.001) as well as a physical functioning score of ≥ 40 at baseline (p = 0.031) was associated with longer overall survival upon multivariate analysis. Upon multivariate analysis of baseline scores associated with treatment duration, only social functioning (p = 0.049) was associated with longer survival.  Changes in HRQOL scores in 12 months prior to death: KW Test, MWU Test: 0 vs 3 months, 0 vs 6 months, 0 vs 9 months, 0 vs 12 months, 3 vs 6 months, 3 vs 9 months, 3 vs 12 months, 6 vs 9 months, 6 vs 12 months, 9 vs 12 months   - Physical Functioning: 0, 0.001, 0.001, 0, 0.007, 0.638, 0.192, 0.312, 0.683, 0.493, 0.606 - Role Physical: 0.008, 0.062, 0.009, 0.032, 0.003, 0.276, 0.539, 0.09, 0.647, 0.463, 0.335 - Bodily Pain: 0.981, 0.829, 0.717, 0.714, 0.967, 0.636, 0.596, 0.699, 0.935, 0.962, 0.973 - General Health: 0.012, 0.1, 0.014, 0.002, 0.088, 0.437, 0.086, 0.508, 0.235, 0.888, 0.657 - Vitality: 0, 0.006, 0.012, 0, 0.002, 0.871, 0.095, 0.038, 0.076, 0.03, 0.212 - Social Functioning: 0.798, 0.67, 0.305, 0.488, 0.376, 0.533, 0.677, 0.556, 0.92, 0.737, 0.781 - Role Emotional: 0.442, 0.381, 0.114, 0.17, 0.248, 0.519, 0.565, 0.583, 0.947, 1, 0.832 - Mental Health: 0.048, 0.044, 0.12, 0.007, 0.117, 0.921, 0.291, 0.486, 0.241, 0.312, 0.891   Changes in HRQOL scores in patients who survived >1 year: Friedman Test, WTS Test: 0 vs 3 months, 0 vs 6 months, 0 vs 9 months, 0 vs 12 months, 3 vs 6 months, 3 vs 9 months, 3 vs 12 months, 6 vs 9 months, 6 vs 12 months, 9 vs 12 months   - Physical Functioning: 0.075, 0.164, 0.189, 0.075, 0.624, 0.767, 1, 0.13, 1, 0.071, 0.109 - Role Physical: 0.549, 0.575, 0.834, 0.505, 0.505, 0.953, 0.397, 0.929, 0.14, 0.211, 0.284 - Bodily Pain: 0.338, 0.213, 0.286, 0.583, 0.272, 0.859, 0.508, 0.065, 0.415, 0.031, 0.508 - General Health: 0.579, 0.366, 0.889, 0.814, 0.248, 0.475, 0.556, 0.814 , 1, 0.114, 0.1 - Vitality: 0.262, 0.059, 0.158, 0.31, 0.824, 0.583, 0.878, 0.133, 0.656, 0.05, 0.049 - Social Functioning: 0.677, 0.397, 0.674, 0.799, 0.476, 0.759, 0.362, 0.507, 0.866, 0.05, 0.31 - Role Emotional: 0.285, 0.283, 0.638, 0.894, 0.767, 0.779, 0.753, 0.063, 0.933, 0.314, 0.181 - Mental Health: 0.486, 0.126, 0.207, 1, 0.576, 0.799, 0.327, 0.278, 0.248, 0.114, 0.255   The domains bodily pain, general health, social functioning and mental health did not show significant changes across the time measured. Domains relating to physicality such as physical functioning, role physical and vitality saw significant decline nearing death. | | | | | | | | | | |  |
| **2017** | | **Bruix et al.** | Self-Administered Questionnaires  (FACT-HEP + FACT-G + EQ5D) | | Baseline, end of trial | | Yes | | Regorafenib | | No | |  |  |
|  |  |  | LS Mean time adjusted AUC: Regorafenib AUC (95%CI), Placebo AUC (95%CI), p-value   - EQ-5D index: 0.76 (0.75-0.78), 0.77 (0.75-0.79), p = 0.4695 - EQ-5D VAS: 71.68 (70.46-72.90), 73.45 (71.84-75.06), p = 0.0558 - FACT-General (FACT-G): 75.14 (74.12-76.16), 76.55 (75.20-77.90), p = 0.0698 - FACT-Hep total: 129.31 (127.84-130.79), 133.17 (131.21-135.12), p = 0.0006   No clinically meaningful differences were noted between the regorafenib and placebo groups in HRQOL. Overall changes from baseline in EQ-5D and FACT-Hep were similar in the two groups.  In the least-mean-squares time adjusted analysis of EQ-5D and Fact-hep, the scores were lower in the regorafenib group than the placebo group  (p = 0.0006) but the minimally important thresholds for the differences were not met. | | | | | | | | | | |  |
| **2017** | | **Chau et al.** | Self-Administered Questionnaires  (FHSI-8 + EQ5D) | | Baseline, 6 weeks then every 12 weeks thereafter | | Yes | | Ramucirumab | | No | |  |  |
|  |  |  | FHSI-8 total scores: ITT Ramucirumab mean (SD) Placebo mean (SD), AFP≥400 ng/mL Ramucirumab mean (SD) Placebo mean (SD)   - Baseline: 26.17 (4.898) 26.70 (4.647), 25.84 (5.030) 25.92 (4.978) - Cycle 4: 25.98 (4.251) 26.28 (5.313), 25.86 (4.196) 25.52 (4.846) - Cycle 10: 26.30 (4.731) 27.54 (4.043), 25.82 (4.807) 27.80 (4.686) - Cycle 16: 26.50 (4.861) 28.50 (3.050), 25.24 (5.389) 28.29 (3.147) - End of treatment: 23.66 (5.964) 24.28 (6.552), 23.80 (5.685) 22.58 (6.560) - End of treatment change from baseline -2.44 (5.561) -2.86 (5.618) (p = 0.3722), -2.21 (5.627) -3.73 (5.875) (p = 0.0381)   Upon comparison of the ITT group who received ramucirumab and placebo, no significant difference was found (p = 0.3722), however when comparing patients who had AFP≥400 ng/mL, the ramucirumab group had a significantly (p = 0.0381) smaller deterioration in FHSI-8 total score.  Upon analysis of change in tumour size and FHSI-8 scores, there was a linear relationship in both ITT (slope = 0.1312, p = 0.0023) and AFP≥400 ng/mL (slope = 0.1979, p = 0.1979) patients showing that more tumour progression may lead to a higher deterioration in HRQOL.  In the ITT population there was no significant difference in the time to initial deterioration in FHSI-8 score between treatment and placebo (HR 95% CI, p-value)) (1.037 (0.802-1.341), p = 0.782). In AFP≥400 ng/mL patients, the time to first deterioration approached significance (HR 95% CI, p-value)) (0.690 (0.470-1.014), p = 0.054).  FHSI-8 Time to Deterioration in AFP≥400 ng/mL patients: HR (95% CI)   - Lack of Energy: 0.884 (0.605-1.292) - Nausea: 0.931 (0.586-1.479) - Pain: 0.641 (0.427-0.962) - Losing Weight: 0.771 (0.467-1.273) - Pain in Back: 0.743 (0.486-1.137) - Fatigue: 0.775 (0.525-1.142) - Jaundice: 0.963 (0.461-2.011) - Discomfort or Pain in Stomach: NR Only the pain domain of FHSI-8 showed a significant reduction (HR 95% CI) (0.427-0.962).   EQ-5D summary index: ITT Ramucirumab mean (SD) Placebo mean (SD), AFP≥400 ng/mL Ramucirumab mean (SD) Placebo mean (SD)  Change from baseline score (EQ-5D)   - Cycle 4: -0.038 (0.189) -0.046 (0.245), -0.031 (0.208) -0.071 (0.277) - Cycle 10: -0.054 (0.212) 0.003 (0.148), -0.024 (0.206) 0.034 (0.191) - Cycle 16: -0.062 (0.214) -0.012 (0.085), -0.028 (0.192) -0.009 (0.114) - End of treatment: -0.129 (0.290) -0.144 (0.280), -0.120 (0.320) -0.191 (0.297)   Change from baseline score (EQ-5D VAS)   - Cycle 4: -1.2 (16.37) -1.3 (18.39), -2.4 (19.91) -4.0 (18.59) - Cycle 10: -2.0 (13.46) 3.8 (15.58), -0.8 (17.24) 8.0 (11.60) - Cycle 16: -0.2 (17.16) 3.6 (13.41), -0.2 (23.86) 7 7.6 (10.50) - End of treatment: -10.8 (19.18) -9.0 (18.26), -11.8 (21.27) -11.9 (18.42)   P-values for EQ-5D results were not reported, but there is minimal difference in ramucirumab vs placebo for both patient groups. | | | | | | | | | | |  |
| **2017** | | **Meyer et al.** | Self-Administered Questionnaire  (QLQ-C30 and QLQ-HCC18) | | Baseline, week 10 and every 6 weeks thereafter.  Median follow up time was 620.0 days (95% CI 572-784) | | Yes | | Sorafenib | | Sorafenib + TACE vs Placebo + TACE | |  |  |
|  |  |  | After 360 days the QLQ-C30 domains social (p = 0.045) and role functioning (p = 0.05) had significantly worsened by up to 6% in the sorafenib group. Diarrhoea (p = 0.0095) and appetite loss (p = 0.0018) domains also significantly worsened by up to 13% and 10% respectively. Using QLQ-HCC18, the nutritional problem domain worsened by up to 7% (p = 0.0084) in the sorafenib group. Notably, the sorafenib group encountered well documented adverse effects such as stomatitis, palmar-plantar erythrodysesthesia and diarrhoea, which could have led to worse HRQOL outcomes. | | | | | | | | | | |  |
| **2018** | | **Chow et al.** | Self-Administered Questionnaire (EQ-5D) | | Baseline, every month during treatment and 6-month intervals thereafter | | No | | Sorafenib | | Sorafenib vs TARE | |  |  |
|  |  |  | Interaction between treatment (TARE vs Sorafenib) and time: Treatment (p-value), Time (p-value), Treatment x Time (p-value) (where Time is in months)   - EQ-5D Index mean for ITT population: 0.022 (p = 0.43), -0.0018 (p = 0.09), -0.0019 (p = 0.19) - EQ-5D Index mean for Treated population: 0.0387 (p = 0.20), -0.0021 (p = 0.0464), -0.0016 (p = 0.30)   There were no statistically significant differences found between the group who received TARE and the group who received sorafenib in either population. | | | | | | | | | | |  |
| **2019** | | **Eilard et al.** | Self-Administered Questionnaires  (QLQ-C30 and QLQ-HCC18) | | Baseline, 1, 4 weeks and every 4 weeks thereafter. | | No | | Sorafenib | | No | |  |  |
|  |  |  | A significant worsening in the QLQ-C30 domains: nausea and vomiting (p = 0.043), appetite loss (p = 0.008) and pain (p = 0.045) was observed. A similar but non-significant worsening was seen in the domains social functioning, cognitive functioning, physical functioning, role functioning and QOL. No significant changes to QLQ-HCC18 domains were seen, but a similar non-significant worsening was observed in the domains fatigue and fever and pain. The nutrition domain saw the highest symptom burden at 4 weeks post treatment, while the diarrhoea domain saw the highest symptom burden at 8 weeks post treatment. | | | | | | | | | | |  |
| **2020** | **Baldan Ferrari et al.** | | Self-Administered Questionnaires  (QLQ-C30 and QLQ-HCC18) | | Baseline, 1, 4 weeks and every 4 weeks thereafter. | | No | | Sorafenib | | No | |  |  |
|  |  |  | A significant worsening in the QLQ-C30 domains emotional functioning (p = 0.0313) and pain (p = 0.0313) was found after the first treatment cycle. Patients who reported pain had a statistically significant worsening in the QLQ-HCC18 pain domain (p = 0.0449) and patients who presented with palmar-plantar erythrodysesthesia had a significant worsening in the body image domain (p = 0.0442). | | | | | | | | | | |  |
| **2020** | | **Finn et al.** | | Self-Administered Questionnaires  (QLQ-C30) | | Baseline, Day 1 of each treatment cycle and every 3 months for one year after treatment cessation. | | No | | Atezolizumab/Bevacizumab vs Sorafenib | | No |  |  |
|  |  |  |  | Treatment with atezolizumab-bevacizumab delayed time to deterioration compared to sorafenib  QLQ-C30 Score time to Deterioration: Atezolizumab-Bevacizumab time (months), Sorafenib time (months), HR, (95%CI)   - QLQ-C30 Score: 11.2, 3.6, 0.63, (0.46-0.85) - Physical Functioning: 13.1, 4.9, 0.53, (0.39-0.73) - Role Functioning: 9.1, 3.6, 0.62, (0.46-0.84) | | | | | | | | | |  |
| **2020** | | **Muszbek et al.** | | Self-Administered Questionnaire  (QLQ-C30) | | NR | | No | | Sorafenib | | Sorafenib vs TACE |  |  |
|  |  |  |  | Patients who received radiation therapy in the SARAH trial had significantly improved global health status in the QLQ-C30 questionnaire (p = 0.0048) when compared to the sorafenib group. Radiation therapy was found to cost less than sorafenib (£29,530 vs £30,957) yet result in higher total QALYs (1.982 vs 1.381). This benefit was mostly because of progression free survival. | | | | | | | | | |  |
| **2020** | | **Zhu et al.** | | Self-Administered Questionnaires  (FHSI-8 and EQ-5D) | | Baseline, 6 weeks following first treatment and every 12 weeks thereafter in REACH, Baseline and every 6 weeks in REACH2 | | Yes | | Ramucirumab | | No |  |  |
|  |  |  |  | In the pooled population with AFP≥400ng/mL, the median time to deterioration in FHSI-8 total score was prolonged with ramucirumab compared to placebo (3.3 vs 1.9 months; HR 0.725; (95% CI 0.559-0.941), p=0.0152). There was no significant difference in time to deterioration of EQ-5D score between ramucirumab and placebo groups (p=0.2382).  Baseline Score: Ramucirumab mean (SD), Placebo mean (SD)   - FHSI-8 Pooled: 26.44 (4.82), 26.29 (5.03) - FHSI-8 REACH AFP≥400ng/mL: 25.84 (5.03), 25.92 (4.98) - FHSI-8 REACH-2: 26.80 (4.66), 26.78 (5.08) - EQ-5D Pooled: 0.784 (0.190), 0.806 (0.193) - EQ-5D REACH AFP≥400ng/mL: 0.778 (0.215), 0.807 (0.202) - EQ-5D REACH-2: 0.787 (0.174), 0.806 (0.182)   Pooled FHSI-8 Score Time to Deterioration: Ramucirumab time (months), Placebo (Months), HR, 95% CI, p-value   - Lack of energy: 2.8, 2.2, 0.942 (0.724-1.225), p=0.6005 - Nausea: 7.8, 4.4, 0.821 (0.589-1.145), p=0.2287 - Pain: 4.2, 2.6, 0.769 (0.588-1.005), p=0.0248 - Weight loss: 6.0, 3.3, 0.699 (0.505-0.969), p=0.0231 - Back pain: 5.7, 2.8, 0.668 (0.497-0.899), p=0.0044 - Fatigue: 2.9, 1.9, 0.813 (0.626-1.056), p=0.0868 - Jaundice: N/A, N/A, 0.725 (0.411-1.278), p=0.2394 - Discomfort/pain in stomach: 3.0, 3.1, 1.017 (0.767-1.349), p=0.8603   REACH-2 FHSI-8 Score Time to Deterioration: Ramucirumab time (months), Placebo (Months), HR, 95% CI, p-value   - Lack of energy: 2.8, 3.0, 1.122 (0.759-1.659), p=0.5798 - Nausea: 8.2, 4.4, 0.809 (0.498-1.314), p=0.4144 - Pain: 4.2, 2.9, 0.913 (0.613-1.361), p=0.5837 - Weight loss: 6.9, 3.3, 0.722 (0.459-1.134), p=0.1579 - Back pain: 6.5, 2.9, 0.542 (0.360-0.838), p=0.0052 - Fatigue: 2.9, 2.2, 0.832 (0.576-1.202), p=0.3044 - Jaundice: N/A, N/A, 0.591 (0.237-1.473), p=0.2538 - Discomfort/pain in stomach: 4.1, 4.4, 0.963 (0.697-1.455), p=0.8422 | | | | | | | | | |  |
| **2021** | | **Galle et al.** | | Self-Administered Questionnaires  (QLQ-C30 and QLQ-HCC18) | | Baseline and every 3 months for 1 year after treatment.  After treatment follow up time: Median (IQR)  8.6 Months (6.2-10.8) | | No | | Atezolizumab/Bevacizumab vs Sorafenib | | No |  |  |
|  |  |  |  | QLQ-C30 score deterioration: Atezolizumab/Bevacizumab (events (%) median time (months) (95% CI), Sorafenib (events (%)), median time (months) (95% CI), Hazard Ratio (95% CI)   - Fatigue: 49% 5.7 (4.3-7.1), 47% 2.1 (1.5-4.8), 0.61 (0.46-0.81) - Pain: 39% 9.7 (7.2-NE), 46% 2.8 (2.1-4.3), 0.46 (0.34-0.62) - Appetite Loss: 30% NE (NE), 33% 7.6 (3.5-NE), 0.57 (0.40-0.81) - Diarrhoea: 18% NE (NE), 38% 4.4 (3.5-5.6), 0.23 (0.16-0.34) - Nausea and Vomiting: 16% NE (NE), 24% NE (5.7-NE), 0.39 (0.26-0.60) - Dyspnoea: 24% NE (13.2-NE), 26% NE (6.3-NE), 0.54 (0.37-0.79) - Insomnia: 25% NE (13.8-NE), 24% NE (7.0-NE), 0.67 (0.46-0.99) - Constipation: 22% NE (13.9-NE), 19% NE (NE), 0.71 (0.46-1.08) - Financial Difficulties: 18% NE (NE), 12% NE (NE), 0.94 (0.55-1.60) - Emotional Functioning: 18% NE (NE), 23% NE (6.7-NE), 0.47 (0.31-0.71) - Social Functioning: 36% NE (8.3-NE), 33% NE (3.5-NE), 0.71 (0.51-0.98) - Cognitive Functioning: 31% NE (10.6-NE), 33% 6.7 (4.2-NE), 0.56 (0.40-0.79)   QLQ-HCC18 score deterioration: Atezolizumab/Bevacizumab (events (%) median time (months) (95% CI), Sorafenib (events (%)), median time (months) (95% CI), HR (95% CI)   - Fatigue: 46% 5.7 (4.3-9.0), 48% 2.1 (1.6-2.8), 0.60 (0.45-0.80) - Pain: 30% NE (NE), 30% 9.8 (4.3-NE), 0.65 (0.46-0.92) - Jaundice: 38% 10.6 (6.9-NE), 31% 6.5 (5.6-NE), 0.76 (0.55-1.07) - Abdominal Swelling: 21% NE (NE), 21% NE (NE), 0.57 (0.37-0.86) - Body Image: 40% 9.0 (6.4-NE), 37% 4.3 (2.8-NE), 0.71 (0.52-0.97) - Fever: 24% NE (NE), 22% NE (NE), 0.71 (0.47-1.06) - Nutrition: 30% NE (NE), 32% 6.9 (3.6-NE), 0.56 (0.40-0.79) - Sex Life: 21% NE (NE), 18% NE (NE), 0.82 (0.53-1.26)   Atezolizumab/Bevacizumab was associated with a significant reduced risk of deterioration in the QLQ-C30 domains (HR (95% CI)): fatigue (0.61 (0.46-0.81)), pain (0.46 (0.34-0.62)), appetite loss (0.57 (0.40-0.81)), diarrhoea (0.23 (0.16-0.34)), nausea and vomiting (0.39 (0.26-0.60)), dyspnoea (0.54 (0.37-0.79)), insomnia (0.67 (0.46-0.99)), emotional functioning (0.47 (0.31-0.71)), social functioning (0.71 (0.51-0.98)) and cognitive functioning (0.56 (0.40-0.79)).   QLQ-HCC18 domains that saw a significant reduced risk of deterioration were (HR (95% CI)): fatigue (0.60 (0.45-0.80)), pain (0.65 (0.46-0.92)), abdominal swelling (0.57 (0.37-0.86)), body image (0.71 (0.52-0.97)) and nutrition (0.56 (0.40-0.79)).  QLQ-C30 change from baseline: Atezolizumab/Bevacizumab mean (SD), Sorafenib mean (SD)   - QOL: -3.29 (17.56), -5.83 (20.63) - Role Functioning: -4.02 (19.42), -9.76 (21.33) - Physical Functioning: -3.77 (12.82), -7.60 (15.54)   Although the significance of the change from baseline is not reported, the reduction in HRQOL scores is considerably less when comparing Atezolizumab/Bevacizumab vs sorafenib. | | | | | | | | | |  |
| **2021** | | **Kudo et al.** | | Self-Administered Questionnaires  (FACT-Hep and EQ-5D-3L) | | Baseline, and the first day of every cycle thereafter. Each cycle was 2 weeks apart. | | Yes | | Nivolumab | | No |  |  |
|  |  |  |  | Mixed model with repeated measures analyses were conducted for EQ-5D-3L and FACT-Hep.  EQ-5D visual analogue scale score (LS -3.2, 95%CI (-8.5-2.0)) remained stable over time with no meaningful decline observed through week 36. The overall utility index was similar, with no clinically meaningful decline observed through week 28  (LS -0.063, 95%CI (-0.118--0.007)). FACT-Hep also showed similar results with no clinically meaningful decline observed in 91.7% of evaluable timepoints. The LS means for FACT-Hep and HCS were -7.9 and -3.6 months respectively. | | | | | | | | | |  |
| **2021** | | **Pereira et al.** | | Self-Administered Questionnaires  (QLQ-C30) | | Baseline, 1-, 3- months post treatment and every 3 months thereafter  After treatment: Median (IQR) (months)  TARE: 29.2 (21.9-34.8)  Sorafenib: 29.6 (21.4-35.1) | | Yes | | Sorafenib | | Sorafenib vs TARE |  |  |
|  |  |  |  | QLQ-C30 Scores: TARE: baseline 1 month 3 months 6 months 9 months 12 months, Sorafenib: baseline 1 month 3 months 9 months 6 months   - Fatigue: 29.9 40.4 43.8 37.3 32.7 35.6, 32.9 52 46.6 47.2 43.2 41.3 - Pain: 19.4 23.7 4.3 21.8 13.7 22, 20.8 33.9 28.7 29.1 29.3 21.9 - Appetite Loss: 18.9 24.9 29.6 20.1 11.8 12.5, 18.1 43.2 38.9 37.2 37.4 36.5 - Diarrhoea: 12.7 12.9 15.6 14.4 14.7 18.1, 15.1 34.3 44.2 51 47.6 61.5 - Nausea and Vomiting: 4.5 10.3 12.4 6.2 3.9 5.1, 6.3 11.8 10.2 9.7 16 13.5 - Dyspnoea: 27.5 29.6 30.3 24.9 25.5 31.9, 23.9 32.9 32.5 34.5 27.2 34.4 - Insomnia: 27 30.8 32.2 36.7 28.4 30.6, 33.5 31.9 32.8 33.7 29.8 25 - Constipation: 13.9 17 18.2 16.7 13.7 18.8, 17.1 21.6 17.4 16.3 13.6 14.6 - Financial Difficulties: 12.8 9.7 11.1 14.1 12.1 9.1, 10.3 12.9 17.6 15.9 22.4 22.9 - Emotional Functioning: 77.4 77.1 78.5 79.8 84.1 83, 71 71.4 70.7 71.5 70.9 72.6 - Social Functioning: 88.1 79.4 79.4 88.1 83.3 86.8, 84.9 70.5 66.8 73.6 66.3 65.1 - Cognitive Functioning: 85.7 85.4 83.5 83.9 82.4 81.3, 84.8 80.8 81.3 84.1 78.6 79.2 - Physical Functioning: 83.4 76.2 73.4 78.9 81 73.2, 82.6 70.7 73.6 73.6 74.1 74.6 - Role Functioning: 83.6 70.7 73.3 78.7 83.3 81.9, 80 63.1 64.3 69.8 67 73.4   Interaction between treatment (TARE vs Sorafenib) and time: Treatment (p-value), Time (p-value), Treatment x Time (p-value)   - Fatigue: (p < 0.0001) (p < 0.0001), (p = 0.10) - Pain: (p = 0.030), (p < 0.0001), (p = 0.16) - Appetite Loss: (p < 0.0001), (p < 0.0001), (p = 0.16) - Diarrhoea: (p < 0.0001), (p < 0.0001), (p < 0.0001), - Nausea and Vomiting: (p = 0.05), (p < 0.0001), (p = 0.07) - Dyspnoea: (p = 0.30), (p = 0.003), (p = 0.12) - Insomnia: (p = 0.70), (p = 0.40), (p = 0.66) - Constipation: (p = 0.72), (p = 0.31), (p = 0.70) - Financial Difficulties: (p = 0.27), (p = 0.003), (p = 0.14) - Emotional Functioning: (p = 0.0004), (p = 0.94), (p = 0.26) - Social Functioning: (p < 0.0001), (p < 0.0001), (p = 0.003) - Cognitive Functioning: (p = 0.21), (p = 0.001), (p = 0.75) - Physical Functioning: (p = 0.22), (p < 0.0001), (p = 0.32) - Role Functioning: (p = 0.003), (p < 0.0001)**,** (p = 0.23) - QOL: (p = 0.006), (p < 0.0001), (p = 0.12)   There was a significant treatment effect for the domains: fatigue (p < 0.0001), pain (p = 0.030), appetite loss (p < 0.0001), diarrhoea (p < 0.0001), nausea and vomiting (p = 0.05), emotional functioning (p = 0.0004), social functioning (p < 0.0001), role functioning (p = 0.003) and QOL (p = 0.006). Of these, the only domains to show a significant treatment by time interaction were diarrhoea (p < 0.0001), nausea and vomiting (p = 0.07) and social functioning (p = 0.003).  Upon analysis of the QOL responses, there was a statistically significant worse responses (TARE % vs Sorafenib % (p-value)) in the domains: nausea and vomiting (47.2% vs 33.6% (p = 0.012), appetite loss (69.1% vs 41.0% (p < 0.0001), diarrhoea (70.8% vs 26.4% (p < 0.0001)) and social functioning (66.5% vs 46.7% (p = 0.004))  QLQ-C30 domain median time (months) to deterioration: TARE median (95% CI), Sorafenib median (95% CI), Hazard Ratio (95% CI)   - Emotional Functioning: 3.98 (3.75-4.40), 3.45 (3.19-5.59), 1.03 (0.81-1.31) - Social Functioning: 3.98 (3.71-4.76), 2.73 (2.14-3.09), 0.60 (0.47-0.76) - Cognitive Functioning: 3.88 (3.58-4.24), 3.19 (2.92-3.45), 0.86 (0.68-1.10) - Physical Functioning: 3.88 (3.48-4.37), 2.73 (2.07-3.12), 0.77 (0.60-0.97) - Role Functioning: 3.68 (2.69-4.34), 2.56 (1.97-3.02), 0.72 (0.57-0.92) - QOL: 3.94 (3.71-4.34), 2.60 (1.97-3.02), 0.59 (0.47-0.76)   TARE treatment was associated with a significant reduced risk in deterioration in the domains social functioning, physical functioning, role functioning and QOL. Although the other domains were not significant, the time to deterioration on sorafenib was still shorter. Overall TARE treatment displayed a reduced risk in time to deterioration of QOL compared to sorafenib. | | | | | | | | | |  |
| **2021** | | **Ryoo et al.** | | Self-Administered Questionnaire (QLQ-C30 and QLQ-HCC18) | | Baseline, at weeks 2,3,4,6,9,12,18 and then every 9 weeks until 1 year or end of treatment. | | Yes | | Pembrolizumab | | No |  |  |
|  |  |  |  | LS mean change from baseline to week 12 in QLQ-HCC18 was similar between pembrolizumab and placebo groups.  QLQ-C30 QOL Scores:   - Baseline (Pembrolizumab mean (SD), Placebo mean (SD)): 70.4 (20.1), 68.9 (21.5) - Week 12 (Pembrolizumab mean (SD), Placebo mean (SD)): 69.7 (19.7), 70.0 (19.6) - Change from baseline to Week 12 (LS Mean, 95%CI): -3.0, (-5.5, -0.5), p=0.573   The proportion of patients reporting improvement, stable or deterioration in QOL were generally similar between pembrolizumab and placebo groups. The change from baseline to week 12 in QLQ-C30 were similar in both functional and symptom domain scores between pembrolizumab and placebo arms.  Time to deterioration was not significantly different between the pembrolizumab and placebo groups  QLQ-HCC18 Time to Deterioration Pembrolizumab vs Placebo: HR, 95%CI, p-value   - Abdominal Swelling: 1.08, 0.76-1.54, p=0.6552 - Fatigue: 0.92, 0.71-1.20, p=0.2795 - Pain: 0.97, 0.74-1.27, p=0.4078   Generally, there was no significant difference for HRQOL scores between pembrolizumab and placebo groups.  LS Mean QLQ-C30 Score change from Baseline: Pembrolizumab (95%CI), Placebo (95%CI)   - Global Health Status/QOL: -3.21 (-5.56 - -0.43), -1.93 (-5.24 - 1.60) - Physical Functioning: -5.24 (-7.59 - -3.10), -3.64 (-6.84 - -0.32) - Role Functioning: -7.06 (-10.27 - -3.96), -2.89 (-7.38 - 1.82) - Emotional Functioning: -0.86 (-3.10 - 1.60), 0.86 (-1.82 - 3.96) - Cognitive Functioning: -4.28 (-6.52 - -2.67), -2.57 (-6.52 - 0.75) - Social Functioning: -3.64 (-5.88 - -0.96), -0.96 (-4.71 - 2.99)   LS Mean QLQ-C30 Symptom Scale change from Baseline: Pembrolizumab (95%CI), Placebo (95%CI)   - Fatigue: 3.50 (0.48 - 6.61), 2.12 (-2.03 - 6.26) - Nausea and Vomiting: 1.43 (-0.22 - 3.33), 0.65 (-1.86 - 3.15) - Pain: 4.71 (1.68 - 7.56), 3.93 (-0.13 - 7.90) - Dyspnoea: 3.24 (-0.13 - 6.70), 4.71 (0.22 - 9.11) - Insomnia: 0.39 (-3.33 - 3.93), -2.03 (-6.78 - 3.15) - Appetite Loss: 1.60 (-1.77 - 4.97), -0.39 (-5.05 - 3.93) - Constipation: 0.73 (-2.38 - 3.67), 0.22 (-3.93 - 4.19) - Diarrhoea: -1.25 (-3.76 - 1.25), -2.12 (-5.40 - 1.34) - Financial Difficulties: 1.17 (-1.86 - 4.45), -0.04 (-4.36 - 4.28)   LS Mean QLQ-HCC18 Scale change from Baseline: Pembrolizumab (95%CI), Placebo (95%CI)   - Fatigue-HCC: 3.01 (-0.04 - 5.90), 1.44 (-2.68 - 5.15) - Body Image: 3.92 (1.11 - 6.72), 2.27 (-1.77 - 6.23) - Jaundice: 4.99 (2.60 - 7.38), 1.44 (-1.86 - 4.74) - Nutrition: 2.52 (0.45 - 4.66), 0.95 (-2.10 - 3.67) - Pain-HCC: 3.67 (1.03 - 6.31), 4.91 (1.36 - 8.45) - Fever: 1.53 (-0.37 - 3.26), 0.54 (-2.10 - 2.76) - Abdominal Swelling: 3.01 (-0.29 - 6.31), 2.76 (-1.69 - 7.30) - Sex Life: 1.77 (-2.27 - 5.81), 1.44 (-4.00 - 7.05) | | | | | | | | | |  |
| **2021** | | **Vogel et al.** | | Self-Administered Questionnaire (QLQ-C30 and QLQ-HCC18) | | Baseline, day 1 of each post baseline cycle and end of treatment. Each cycle was 28 days | | Yes | | Sorafenib vs Lenvatinib | | No |  |  |
|  |  |  |  | QLQ-C30 Score: Lenvatinib baseline (mean (SD)) LS mean score, Sorafenib baseline (mean (SD)) LS mean score, Difference in LS Mean score (95%CI), Time to Deterioration HR (95%CI)   - Global health status/QOL: 67.06 (20.47) 60.04, 67.57 (20.52) 60.78, −0.75 (−3.10-1.61), 0.89 (0.73-1.09) - Physical functioning: 84.80 (16.29) 74.71, 85.63 (15.62) 73.55, 1.17 (−1.74 - 4.07), 0.88 (0.72-1.07) - Role functioning: 85.41 (21.23) 72.35, 85.79 (22.03) 70.69, 1.66 (−1.62 - 4.95), 0.84 (0.69-1.01) - Emotional functioning: 83.69 (16.94) 79.69, 83.41 (16.80) 78.10, 1.59 (−1.00 - 4.17), 0.83 (0.65-1.06) - Cognitive functioning: 89.48 (14.26) 79.22, 87.36 (17.14) 80.34, −1.13 (−3.70 - 1.45), 0.97 (0.80-1.19) - Social functioning: 82.65 (23.06) 72.51, 82.75 (22.19) 70.58, 1.94 (−1.27 - 5.15), 0.87 (0.71-1.06) - Fatigue 26.35 (22.12) 35.80, 25.26 (21.19) 36.79, −0.99 (−3.99 - 2.02), 0.83 (0.69-0.99) - Nausea and vomiting 4.85 (12.70) 9.42, 5.03 (12.41) 10.08, -0.66 (−2.64 - 1.33), 0.97 (0.75-1.25) - Pain 18.45 (23.38) 26.30, 16.52 (21.92) 27.49, −1.19 (−4.09 - 1.72), 0.80 (0.66-0.96) - Dyspnoea 13.76 (21.37) 19.11, 13.99 (21.56) 20.14, −1.03 (−3.88 - 1.81), 0.82 (0.65-1.04) - Insomnia 19.27 (25.56) 23.94, 20.68 (25.47) 21.77, 2.17 (−0.61 - 4.95), 1.02 (0.80-1.30) - Appetite loss 14.42 (23.75) 27.54, 13.52 (22.90) 27.74, −0.20 (−3.67 - 3.27), 0.89 (0.73-1.08) - Constipation 11.80 (22.33) 14.31, 12.61 (21.68) 11.77, 2.54 (0.23 - 4.85), 1.00 (0.75-1.33) - Diarrhoea 7.74 (16.59) 21.10, 5.87 (13.81) 30.78, −9.67 (−12.87 - −6.48), 0.52 (0.42-0.65) - Financial difficulties 27.80 (31.61) 29.52, 26.65 (31.37) 30.46, −0.94 (−4.43 - 2.54), 0.82 (0.63-1.05)   QLQ-HCC18 Score: Lenvatinib baseline (mean (SD)) LS mean score, Sorafenib baseline (mean (SD)) LS mean score, Difference in LS Mean score (95%CI), HR (95%CI)   - Fatigue: 23.33 (19.72) 31.42, 21.80 (19.52) 33.43, −2.01 (−5.04 - 1.02), 0.88 (0.74-1.06) - Jaundice: 8.44 (13.57) 11.53, 7.84 (12.57) 11.47, 0.06 (−1.83 - 1.94), 0.85 (0.66-1.08) - Body image: 15.12 (18.82) 24.37, 14.16 (18.54) 26.11, −1.38 (−4.36 - 1.60), 0.81 (0.67-0.98), - Nutrition: 14.74 (15.21) 21.99, 12.29 (14.26) 25.03, −3.04 (−5.64 - −0.44), 0.74 (0.60-0.90), - Pain: 13.93 (17.74) 19.63, 14.78 (17.62) 18.77, 0.86 (−1.41 - 3.13), 0.97 (0.80-1.24) - Fever: 5.72 (12.12) 9.29, 5.45 (12.58) 9.39, −0.10 (−1.67 - 1.48), 0.85 (0.67-1.08),0.85 (0.67-1.08) - Sex life: 22.61 (31.60) 31.34, 21.87 (31.26) 31.11, 0.23 (−3.97 - 4.44), 0.89 (0.70-1.12) - Abdominal swelling: 13.53 (21.69) 17.77, 12.56 (20.06) 18.86, −1.09 (−3.92 - 1.73),0.92 (0.71-1.18)   EQ-5D Index: Lenvatinib baseline (mean (SD)) LS mean score, Sorafenib baseline (mean (SD)) LS mean score, Difference in LS Mean score (95%CI), HR (95%CI)   - 0.84 (0.18) 0.73, 0.85 (0.17) 0.72, 0.01 (−0.02 to 0.04), 0.84 (0.70-1.01)   Upon analysis, patients treated with Lenvatinib saw significant improvements in time to deterioration (HR (95% CI) in the domains fatigue (0.83 (0.69-0.99)), pain, (0.80 (0.66-0.96)), diarrhoea (0.52 (0.42-0.65)), body image (0.81 (0.67-0.98)), nutrition (0.74 (0.60-0.90) and the EQ-VAS score (0.83 (0.69-0.99)). No other significant differences in time to deterioration was reported. There were no significant differences in individual domain scores although the LS mean score for diarrhoea was numerically significant in favour of Lenvatinib. The LS mean score for constipation favoured sorafenib. There were no significant differences in global health scores, EQ-VAS score or the EQ-5D index. | | | | | | | | | |  |
| **2021** | | **Woei-A-Jin et al.** | | Self-Administered Questionnaire (QLQ-C30 and QLQ-HCC18) | | Baseline, day 12, day 26 and follow up post treatment. | | Yes | | Dovitinib | | No |  |  |
|  |  |  |  | HRQOL generally decreased during dovitinib treatment.  Fatigue and social functioning worsened on day 12 compared to baseline, (p<0.023) but showed recovery at day 26 and follow up. Global health score had deteriorated significantly (p<0.006) at follow up. Cognitive and emotional functioning scales were not affected by the treatment. | | | | | | | | | |  |
| **2022** | | **Abou Alfa et al.** | | Self-Administered Questionnaires  (QLQ-C30) | | Baseline and every 4 weeks thereafter | | Yes | | STRIDE vs Durvalumab vs Sorafenib | | No |  |  |
|  |  |  |  | Time to Deterioration of Quality of Life According to QLQ-C30 Scores: Number of Events (%), Median time to deterioration in months (95%CI), Hazard ratio vs Sorafenib (95%CI)   - STRIDE: 142 (47.0%), 7.5 (5.82-10.84), 0.76 (0.61-0.96) - Durvalumab: 153 (48.0%), 7.4 (5.68-9.33), 0.77 (0.62-0.96) - Sorafenib: 162 (50.2%), 5.7 (4.80-7.39)   There was prolonged time to deterioration of quality of life for the Durvalumab group compared to the Sorafenib group. | | | | | | | | | |  |
| **2022** | | **Agirrezabal et al.** | | Self-Administered Questionnaires  (QLQ-C30) | | Baseline, 1-, 4- weeks and every 4 weeks thereafter. | | No | | Atezolizumab/Bevacizumab vs Sorafenib | | Atezolizumab/Bevacizumab vs TARE Sorafenib vs TARE |  |  |
|  |  |  |  | Time to deterioration in the SARAH trial was significantly (HR, 95% CI, (p-value) (0.69, 0.54-0.88, (p = 0.003) longer for TARE (6.93 months) vs sorafenib (4.30 months).  Analysis TTD in QOL: Base case analysis median time (months) (95% CI), Sensitivity analysis median time (months) (95% CI)   - Atezolizumab-Bevacizumab: 11.23 (6.15-NE), 11.23 (6.15-NE) - TARE: 8.64 (7.16-19.02), 19.88 (9.59-24.30) - Sorafenib (SARAH): 5.52 (4.21-6.67), 5.52 (3.98-18.70) - Sorafenib (IMbrave150): 3.58 (3.00-7.00), 3.58 (3.00-7.00)   Upon base case analysis, the atezolizumab-bevacizumab had the longest median TTD (11.23 months) while TARE (8.64 months) was second and sorafenib in both SARAH and IMbrave150 trials showed the lowest TTD (5.52 and 3.58 months respectively). The difference between TARE and Atezolizumab/Bevacizumab was not statistically significant (HR, 95% CI, (p-value)) (1.06, 0.75-1.50 (p = 0.725)). Upon sensitivity analysis TARE had the highest median TTD (19.88 months) and there was no statistical difference again between TARE and Atezolizumab/Bevacizumab (HR, 95% CI, (p-value)) (0.66 0.36-1.19 (p = 0.163)). Sorafenib had the shortest TTD in HRQOL, with statistically significant differences in both base case and sensitivity analyses. | | | | | | | | | |  |
| **2022** | | ***Charonpongsuntorn et al.*** | | Self-Administered Questionnaires (QLQ-C30 & QLQ-HCC18) | | Baseline and every 12 weeks theraefter | | Yes | | Atezolizumab/Bevacizumab | | No |  |  |
|  |  |  |  | QLQ-C30 Global Health Status after Atezolizumab/Bevacizumab: Mean, Median (IQR) (Range)   - Baseline: 76.67, 75.00 (30.00) (86.00) - 3 Months: 71.57, 75.00 (24.00) (67.00) - 6 Months: 64.10, 66.50 (22.50) (100.00) - 9 Months: 55.20, 58.50 (36.50) (100.00)   The coefficients at baseline and 3 months were not significantly different (difference, 95%CI) (-5.58, -16.44-5.27).  The coefficients at 6 and 9 months were significantly lower when compared to baseline. (difference, 95%CI) (-17.82, -29.8--5.84) and  (-33.46, -47.83--19.06) respectively. The p-value was not reported.  QLQ-C30 Physical Functioning Domain after Atezolizumab/Bevacizumab: Median (IQR) (Range)   - Baseline: 84.67, 93.41 (25.74) (46.38) - 3 Months: 79.61, 93.20 (32.46) (70.50) - 6 Months: 78.97, 93.14 (25.71) (46.24) - 9 Months 72.5, 76.61 (35.27) (86.55)   The physical function scores were not significantly different from baseline to 3 months (difference, 95%CI) (-5.96, -15.27-3.34) but did significantly decline at 6 and 9 months compared to baseline (difference, 95%CI) (-12.58, -22.87—2.29) and (-23.76, -36.18--11.40) respectively. The p-value was not reported  QLQ-HCC18 Score after Atezolizumab/Bevacizumab: Median (IQR) (Range)   - Baseline: 12.69, 13.01 (17.72) (35.23) - 3 Months: 19.63, 16.72 (19.35) (55.71) - 6 Months: 22.31, 16.50 (19.56) (50.14) - 9 Months: 23.06, 16.74 (29.88) (61.00)   The significance of the steady increase in QLQ-HCC18 score was not reported. | | | | | | | | | | |
| ***2022*** | | ***Freemantle et al.*** | Self-Administered Questionnaires (EQ5D5L) | | Baseline, every 4 weeks until week 25 and then 8 weeks thereafter | | Yes | | Cabozantinib | | No | |  |  |
|  |  |  | EQ5D5L Scores at baseline: Placebo Mean (SD) Median (IQR), Cabozantinib Mean (SD) Median (IQR)   - **Mobility:** 1.39 (0.66) 1.00 (1.00–2.00), 1.53 (0.85), 1.00 (1.00–2.00) - **Self-care:** 1.12 (0.44) 1.00 (1.00–1.00), 1.22 (0.60), 1.00 (1.00–1.00) - **Usual activities:** 1.46 (0.77) 1.00 (1.00–2.00), 1.54 (0.84), 1.00 (1.00–2.00) - **Pain/discomfort:** 1.79 (0.83) 2.00 (1.00–2.00), 1.97 (0.95), 2.00 (1.00–3.00) - **Anxiety/depression:** 1.47 (0.65) 1.00 (1.00–2.00), 1.60 (0.81), 1.00 (1.00–2.00) - **Utility:** 0.81 (0.17) 0.84 (0.74–1.00), 0.77 (0.21), 0.80 (0.68–0.88)   At baseline, mean EQ5D5L scores were higher for the cabozantinib group compared to the placebo group.  Difference in EQ5D5L VAS scores by follow up visit, compared to baseline score (Cabozantinib – Placebo)   - **Week 5:** -6.812 (-9.46 - -4.164), p < 0.0001 - **Week 9:** -3.631 (-6.502 - -0.760), p = 0.013 - **Week 13:** -3.141 (-7.041 - 0.759), p = 0.114 - **Week 17:** -5.090 (-9.751 - -0.429), p = 0.032 - **Week 21:** -6.082 (-11.273 - -0.891), p = 0.022 - **Week 25:** -6.120 (-11.590 - -0.649), p = 0.029 - **Week 33:** -0.215 (-6.566 - 6.136), p = 0.947 - **Week 41:** -2.696 (-11.002 - 5.610), p = 0.521 - **Week 49:** -3.876 (-14.050 - 6.298), p = 0.451 - **Week 57:** -1.305 (-14.396 - 11.786), p = 0.842 - **Week 65:** 3.296 (-12.867 - 19.459), p = 0.683 - **Week 73:** -0.422 (-18.426 - 17.582), p = 0.962 - **Week 81:** -0.792 (-18.588 - 17.004), p = 0.927   There were statistically significant differences in favour of the placebo group until week 33.  Difference in EQ5D5L Utility scores by follow up visit, compared to baseline score (Cabozantinib – Placebo)   - Week 5: -0.097 (-0.126 - -0.067), p < 0.0001 - Week 9: -0.051 (-0.083 - -0.018), p = 0.0021 - Week 13: -0.050 (-0.091 - -0.009), p = 0.0181 - Week 17: -0.053 (-0.102 - -0.005), p = 0.0316 - Week 21: -0.057 (-0.110 - -0.004), p = 0.0342 - Week 25: -0.043 (-0.096 - 0.011), p = 0.1165 - Week 33: 0.003 (-0.063 - 0.070), p = 0.9189 - Week 41: -0.021 (-0.117 - 0.074), p = 0.6599 - Week 49: 0.038 (-0.063 - 0.138), p = 0.4589 - Week 57: -0.005 (-0.134 - 0.123), p = 0.9333 - Week 65: 0.045 (-0.150 - 0.241), p = 0.6431 - Week 73: -0.055 (-0.244 - 0.135), p = 0.5599 - Week 81: -0.056 (-0.250 - 0.138), p = 0.5572   There were statistically significant differences in favour of the placebo group until week 21.  EQ5D5L scores at the end of follow up: Cabozantinib Mean (SD), Placebo Mean (SD), Difference (Cabozantinib – Placebo), 95%CI, p-value   - Mobility: 1.89 (0.95), 1.54 (0.81), 1.24, 1.14 - 1.34, <0.0001 - Self-care: 1.45 (0.79), 1.25 (0.62), 1.14, 1.04 - 1.24, 0.0033 - Usual activities: 1.93 (0.95), 1.63 (0.87), 1.20, 1.10 - 1.30, <0.0001 - Pain/discomfort: 2.18 (0.94), 1.93 (0.91), 1.13, 1.06 - 1.21, 0.0005 - Anxiety/depression: 1.62 (0.81), 1.53 (0.72), 1.07, 0.99 - 1.16, 0.1104   At the end of follow up, the mean EQ5D5L score was significantly higher for the Cabozantinib group for the domains mobility(p<0.0001), self-care (p<0.0033), usual activities (p<0.0001) and pain/discomfort (p<0.0005). | | | | | | | | | | |  |
| ***2022*** | | ***Qiao et al.*** | Self-Administered Questionnaires (QLQ-C30) | | Baseline and every 3 months | | Yes | | Pembrolizumab | | No | |  |  |
|  |  |  | All patients completed quality of life assessments and none of them had experienced a deterioration in quality of life in either group. | | | | | | | | | | |  |
| ***2022*** | | ***Yau et al.*** | Self-Administered Questionnaires (Fact-Hep, EQ5D3L) | | Baseline and every cycle thereafter | | Yes | | Nivolumab vs Sorafenib | | No | |  |  |
|  |  |  | Baseline FACT-Hep total score: Mean (SD)   - Nivolumab 140 (21.5) - Sorafenib 140.6 (19.1)   Mean baseline FACE-Hep scores were similar in both treatment groups  LS Mean Scores for FACT-Hep: LS Mean (95%CI)   - Total: 10.1 (7.3–13.0), - Physical well-being: 2.0 (1.4–2.6) - Functional well-being: 2.5 (1.7–3.2)   Adjusted analyses yielded minimally important differences in least squares means favouring nivolumab. No subscales favoured sorafenib  Time to deterioration in FACT-Hep Score: HR (95%CI)   - Total: 0.62 (0.52–0.74), - Physical well-being: 0.62 (0.52–0.74), - Functional well-being: 0.73 (0.61–0.88), - Hepatobiliary cancer sub-scale: 0.57 (0.48–0.69).   Time to treatment deterioration was substantially delayed with nivolumab.  Nivolumab showed improved scores and lengthened time to deterioration compared to sorafenib. | | | | | | | | | | |  |
| ***2022*** | | ***Zou et al.*** | Self-Administered Questionnaires (EORTC QLQ-C30 & QLQ-HCC18) | | Baseline and after treatment | | Yes | | Lenvatinib vs Lenvatinib + PD-1 Inhibitor (Pembrolizumab or Toripalimab) | | No | |  |  |
|  |  |  | QLQC30 scores: Before Lenvantinib mean (SD), After Lenvantinib mean (SD), After Anti PD1 mean (SD), p-value comparing lenvantinib alone vs addition of PD-1 inhibitor   - Physical Function: 86.7 (11.5), 86.2 (4.8), 84.9 (14.7), 0.41 - Role Function: 72.2 (16.7), 71.6 (22.1), 74.7 (21.9), 0.30 - Emotional Function: 91.0 (7.6), 94.1 (7.6), 95.4 (6.7), 0.01 - Cognitive Function: 93.8 (9.4), 94.4 (9.2), 95.8 (8.7), 0.23 - Social Function: 75.3 (18.7), 74.1 (18.9), 76.5 (20.8), 0.45 - Global HRQOL: 54.1 (9.3), 61.8 (11.2), 67.6 (11.6), 0.00 - Fatigue: 16.0 (15.0), 13.9 (14.3), 13.6 (13.5), 0.67 - Nausea/vomiting: 4.3 (8.8), 4.3 (8.8), 1.2 (4.4), 0.06 - Pain: 9.3 (14.1), 13.0 (14.1), 9.9 (15.5), 0.34 - Dyspnoea: 3.7 (10.7), 3.7 (10.6), 3.7 (14.1), 1.00 - Sleep Disturbance: 7.4 (14.1), 4.9 (12.1), 2.5 (8.9), 0.13 - Appetite Loss: 14.8 (16.9), 19.8 (17.9), 12.3 (18.4), 0.17 - Constipation: 2.5 (8.9), 3.7 (10.7), 2.5 (8.9), 0.57 - Diarrhea: 4.9 (15.2), 18.5 (16.8), 12.3 (20.9), 0.01 - Financial Difficulties: 13.6 (16.7), 12.3 (18.8), 13.6 (19.1), 0.75   There were significant improvements after anti-PD1 therapy compared to monotherapy for the emotional function (p = 0.01), global HRQOL (p = 0.00) and diarrhoea (p = 0.01) subscales.  QLQHCC18 scores: Before Lenvantinib mean (SD), After Lenvantinib mean (SD), After Anti PD1 mean (SD), p-value comparing lenvantinib alone vs addition of Anti PD1   - Abdominal swelling: 13.6 (23.1), 14.8 (23.3), 14.8 (19.2), 0.95 - Body image: 0.6 (3.2), 0.6 (3.1), 0.6 (3.2), 1.00 - Jaundice: 0.0 (0.0), 0.6 (3.1), 1.8 (5.3), 0.11 - Pain: 4.9 (10.1), 11.1 (13.9), 5.5 (10.3), 0.01 - Fever: 0.6 (3.2), 1.8 (5.3), 3.7 (7.1), 0.16 - Nutrition: 0.7 (2.8), 0.7 (2.1), 1.0 (2.4), 0.75 - Fatigue: 10.3 (11.5), 11.1 (12.7), 4.9 (7.5), 0.03 - Sex life: 3.7 (14.1), 4.9 (15.2), 0.0 (0.0), 0.14   There were significant improvements after anti-PD1 therapy compared to monotherapy for the pain (p = 0.01) and fatigue (p = 0.03) subscales.  Emotional functioning and overall HRQOL were improved significantly in patients with HCC after initiation of treatment with anti-PD1 antibodies plus lenvatinib. The addition of anti-PD1 therapy reduced the incidence of deterioration on the QLQHCC18 symptom scales when compared to Lenvatinib monotherapy. | | | | | | | | | | |  |
| AFP, Alpha Fetoprotein; AUC, Area under the curve; BSC, best supportive care; CI, confidence interval; EQ-5D, EuroQol-5 Dimension Questionnaire; EQ5D3L, EuroQol-5 Dimension Questionnaire; EQ5D5L, EuroQol-5 Dimension Questionnaire with EQ-VAS, EuroQol-visual analogue scales; FACT, Functional Assessment of Cancer Therapy FACT-G, Functional Assessment of Cancer Therapy - General Questionnaire; FACT-Hep, Functional Assessment of Cancer Therapy-Hepatobiliary Questionnaire; FHSI-8, Functional Assessment of Cancer Therapy Hepatobiliary Cancer Symptom Index - 8; HCS, Hepatobiliary cancer subscale; HR, Hazard Ratio; HRQOL, Health Related Quality of Life; IQR, Interquartile Range; ITT, Intention to Treat; KW, Kruskal Wallis Test; LS, Least-Squares; MWU, Mann-Whitney U Test; NE, Not Evaluated; NR, Not Reported; OR, Odds Ratio; PD-1, programmed cell death protein 1; QALY, quality-adjusted life-year; QLQ-C30, Quality-of-life Questionnaire Core 30; QLQ-HCC18, Quality of Life Questionnaire Hepatocellular Carcinoma 18; QOL, Quality of Life; REACH, Ramucirumab versus placebo as second-line treatment in patients with advanced hepatocellular carcinoma following first-line therapy with sorafenib; SARAH, Sorafenib versus radioembolisation in advanced hepatocellular carcinoma; SD, Standard Deviation; SF-36, medical outcomes survey short-form 36; STRIDE, Single Tremelimumab Regular Interval Durvalumab; TACE, Transcatheter Chemoembolisation; TARE, Transcatheter Radioembolisation; TTD, Time to Deterioration; VAS, Visual Analogue Scale; WTS, Wilcoxon Two Sample Test | | | | | | | | | | | | | |  |
