# Supplementary material for: Hepatocellular Carcinoma and Health-Related Quality of Life: A Systematic Review of Outcomes From Systemic Therapies
Source: Int J Hepatol. 2025 Apr 7;2025:1083642. doi: 10.1155/ijh/1083642 (PMC11996279; doi:10.1155/ijh/1083642)
Supplement: Supporting Information 5 — Table S5: Canada Institute of Health Economics (IHE) quality appraisal checklist for included case series. [file 1083642.f5.docx]

**Table S5: Canada Institute of Health Economics (IHE) quality appraisal checklist for included case series**

**
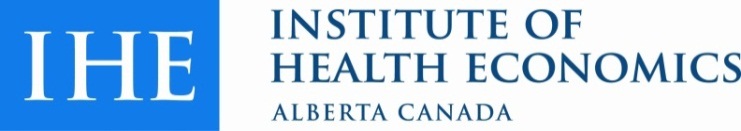
**

**Quality Appraisal Checklist for Case Series Studies***

| **Study objective** | | | |
| --- | --- | --- | --- |
| **1.** | **Was the hypothesis/aim/objective of the study clearly stated?** | Yes | X |
|  |  | Partial | ☐ |
|  |  | No | ☐ |
| **Study design** | | | |
| **2.** | **Was the study conducted prospectively?** | Yes | ☐ |
|  |  | Unclear | ☐ |
|  |  | No | X |
| **3.** | **Were the cases collected in more than one centre?** | Yes | X |
|  |  | Unclear | ☐ |
|  |  | No | ☐ |
| **4.** | **Were patients recruited consecutively?** | Yes | ☐ |
|  |  | Unclear | X |
|  |  | No | ☐ |
| **Study population** | | | |
| **5.** | **Were the characteristics of the patients included in the study described?** | Yes | X |
|  |  | Partial | ☐ |
|  |  | No | ☐ |
| **6.** | **Were the eligibility criteria (i.e. inclusion and exclusion criteria) for entry into the study clearly stated?** | Yes | X |
|  |  | Partial | ☐ |
|  |  | No | ☐ |
| **7.** | **Did patients enter the study at a similar point in the disease?** | Yes | X |
|  |  | Unclear | ☐ |
|  |  | No | ☐ |
| **Intervention and co-intervention** | | | |
| **8.** | **Was the intervention of interest clearly described?** | Yes | X |
|  |  | Partial | ☐ |
|  |  | No | ☐ |
| **9.** | **Were additional interventions (co-interventions) clearly described?** | Yes | ☐ |
|  |  | Partial | ☐ |
|  |  | No | X |

**This checklist should be cited as:** Institute of Health Economics (IHE). Quality Appraisal of Case Series Studies Checklist. Edmonton (AB): Institute of Health Economics; 2014. Available from: <http://www.ihe.ca/research-programs/rmd/cssqac/cssqac-about>

| **Outcome measure** | | | |
| --- | --- | --- | --- |
| **10.** | **Were relevant outcome measures established a priori?** | Yes | ☐ |
|  |  | Partial | ☐ |
|  |  | No | X |
| **11.** | **Were outcome assessors blinded to the intervention that patients received?** | Yes | ☐ |
|  |  | Unclear | ☐ |
|  |  | No | X |
| **12.** | **Were the relevant outcomes measured using appropriate objective/subjective methods?** | Yes | X |
|  |  | Partial | ☐ |
|  |  | No | ☐ |
| **13.** | **Were the relevant outcome measures made before and after the intervention?** | Yes | X |
|  |  | Unclear | ☐ |
|  |  | No | ☐ |
| **Statistical analysis** | | | |
| **14.** | **Were the statistical tests used to assess the relevant outcomes appropriate?** | Yes | X |
|  |  | Unclear | ☐ |
|  |  | No | ☐ |
| **Results and conclusions** | | | |
| **15.** | **Was follow-up long enough for important events and outcomes to occur?** | Yes | X |
|  |  | Unclear | ☐ |
|  |  | No | ☐ |
| **16.** | **Were losses to follow-up reported?** | Yes | X |
|  |  | Unclear | ☐ |
|  |  | No | ☐ |
| **17.** | **Did the study provided estimates of random variability in the data analysis of relevant outcomes?** | Yes | ☐ |
|  |  | Partial | ☐ |
|  |  | No | X |
| **18.** | **Were the adverse events reported?** | Yes | X |
|  |  | Partial | ☐ |
|  |  | No | ☐ |
| **19.** | **Were the conclusions of the study supported by results?** | Yes | X |
|  |  | Unclear | ☐ |
|  |  | No | ☐ |
| **Competing interests and sources of support** | | | |
| **20.** | **Were both competing interests and sources of support for the study reported?** | Yes | ☐ |
|  |  | Partial | ☐ |
|  |  | No | X |

*Note: Assessor(s) may decide to remove from the checklist the items that are not applicable to their project.
